# Systemic blockade of ACVR2B ligands prevents chemotherapy-induced muscle wasting by restoring muscle protein synthesis without affecting oxidative capacity or atrogenes

**DOI:** 10.1038/srep32695

**Published:** 2016-09-26

**Authors:** T. A. Nissinen, J. Degerman, M. Räsänen, A. R. Poikonen, S. Koskinen, E. Mervaala, A. Pasternack, O. Ritvos, R. Kivelä, J. J. Hulmi

**Affiliations:** 1 Department of Biology of Physical Activity, Neuromuscular Research Center, University of Jyväskylä, Jyväskylä, Finland; 2 Wihuri Research Institute and Translational Cancer Biology Program, University of Helsinki, Helsinki, Finland; 3 LIKES Research Center for Sport and Health Sciences, Jyväskylä, Finland; 4 Department of Pharmacology, Faculty of Medicine, University of Helsinki, Helsinki, Finland; 5 Department of Bacteriology and Immunology, Haartman Institute, University of Helsinki, Helsinki, Finland; 6 Department of Physiology, Faculty of Medicine, University of Helsinki, Helsinki, Finland

## Abstract

Doxorubicin is a widely used and effective chemotherapy drug. However, cardiac and skeletal muscle toxicity of doxorubicin limits its use. Inhibiting myostatin/activin signalling can prevent muscle atrophy, but its effects in chemotherapy-induced muscle wasting are unknown. In the present study we investigated the effects of doxorubicin administration alone or combined with activin receptor ligand pathway blockade by soluble activin receptor IIB (sACVR2B-Fc). Doxorubicin administration decreased body mass, muscle size and bone mineral density/content in mice. However, these effects were prevented by sACVR2B-Fc administration. Unlike in many other wasting situations, doxorubicin induced muscle atrophy without markedly increasing typical atrogenes or protein degradation pathways. Instead, doxorubicin decreased muscle protein synthesis which was completely restored by sACVR2B-Fc. Doxorubicin administration also resulted in impaired running performance without effects on skeletal muscle mitochondrial capacity/function or capillary density. Running performance and mitochondrial function were unaltered by sACVR2B-Fc administration. Tumour experiment using Lewis lung carcinoma cells demonstrated that sACVR2B-Fc decreased the cachectic effects of chemotherapy without affecting tumour growth. These results demonstrate that blocking ACVR2B signalling may be a promising strategy to counteract chemotherapy-induced muscle wasting without damage to skeletal muscle oxidative capacity or cancer treatment.

Cancer-related cachexia has been suggested to account for up to 20–30% of all cancer deaths^1^. In addition, decreased skeletal muscle mass is associated with increased toxicity of chemotherapy and impaired prognosis^2^. In contrast, maintenance of skeletal muscle mass predicts better response to treatment and survival^2^,^3^. Therefore, it is crucial to discover and develop effective strategies to counteract chemotherapy-induced muscle loss.

Doxorubicin is a widely used and effective anthracycline chemotherapeutic agent. Some of its most important antineoplastic effects are suggested to include prevention of DNA replication via DNA Topoisomerase II inhibition, DNA damage via formation of reactive oxygen species (ROS) and apoptosis (programmed cell death)^4^. However, doxorubicin has deleterious effects on several tissues other than tumour, which limits its clinical use. Particularly well-known side-effect is doxorubicin-induced cardiotoxicity^4^,^5^. Doxorubicin has also been shown to have adverse effects on skeletal muscle tissue: muscle weakness, fatigue, dysfunction and atrophy have been reported in both humans^4^,^6^ and animals^4^,^7^,^8^,^9^,^10^,^11^ after chemotherapy. The proposed cellular and molecular mechanisms for skeletal muscle toxicity, at least with high doses, include oxidative stress induced by doxorubicin accumulating into skeletal muscle, which may lead to contractile and mitochondrial dysfunction associated with activation of proteolytic and apoptotic signalling pathways^4^,^8^,^9^,^12^. Protein degradation pathways have been extensively studied in different muscle atrophy models and in human diseases^13^. In adults, muscle size is, however, regulated by the balance between protein synthesis and degradation^14^. Currently, the effect of doxorubicin on muscle protein synthesis is unknown.

Muscle size is negatively regulated by myostatin and activins that belong to the TGF-β superfamily of proteins^15^,^16^. They exert their effect through binding to their receptor activin receptor type IIb (ACVR2B)^17^. An often used strategy to prevent muscle loss in animal models is to block these ACVRIIB ligands by administration of a soluble ligand binding domain of ACVR2B fused to the Fc region of IgG (sACVR2B-Fc). This strategy has been shown to increase muscle mass effectively in mice^17^,^18^,^19^. In addition, sACVR2B-Fc treatment has been found to reverse cancer cachexia and prolong survival in different mouse models of cancer cachexia^20^,^21^. However, blocking ACVR2B ligands can also, depending on the context, have adverse effects^22^,^23^,^24^. It is not known whether sACVR2B-Fc administration could prevent doxorubicin-induced muscle atrophy without negatively altering muscle oxidative capacity.

The effects of blocking ACVR2B signalling on muscle growth in healthy mice have been shown earlier by us and others^19^,^25^. In the present study we investigated the effects of systemic doxorubicin administration alone or combined with sACVR2B-Fc treatment on skeletal muscle size and function and the underlying molecular mechanisms. For this purpose, five doxorubicin experiments were performed: 1–2) two four-week experiments, 3) a two-week experiment, 4) an acute 20 h experiment, and 5) a tumour experiment with doxorubicin treatment. The dose of doxorubicin was selected to mimic clinical doses used in humans. These studies demonstrate that doxorubicin induces muscle atrophy that is, at least in part, due to blunted skeletal muscle protein synthesis. We also showed that blocking ACVR2B signalling can counteract chemotherapy-induced muscle loss without further damage to skeletal muscle oxidative capacity or mitochondria. Importantly, sACVR2B-Fc administration did not affect tumour growth or the effect of doxorubicin on tumour growth.

## Results

### sACVR2B treatment prevents doxorubicin-induced skeletal muscle atrophy but not loss of fat

**Figure 1:**
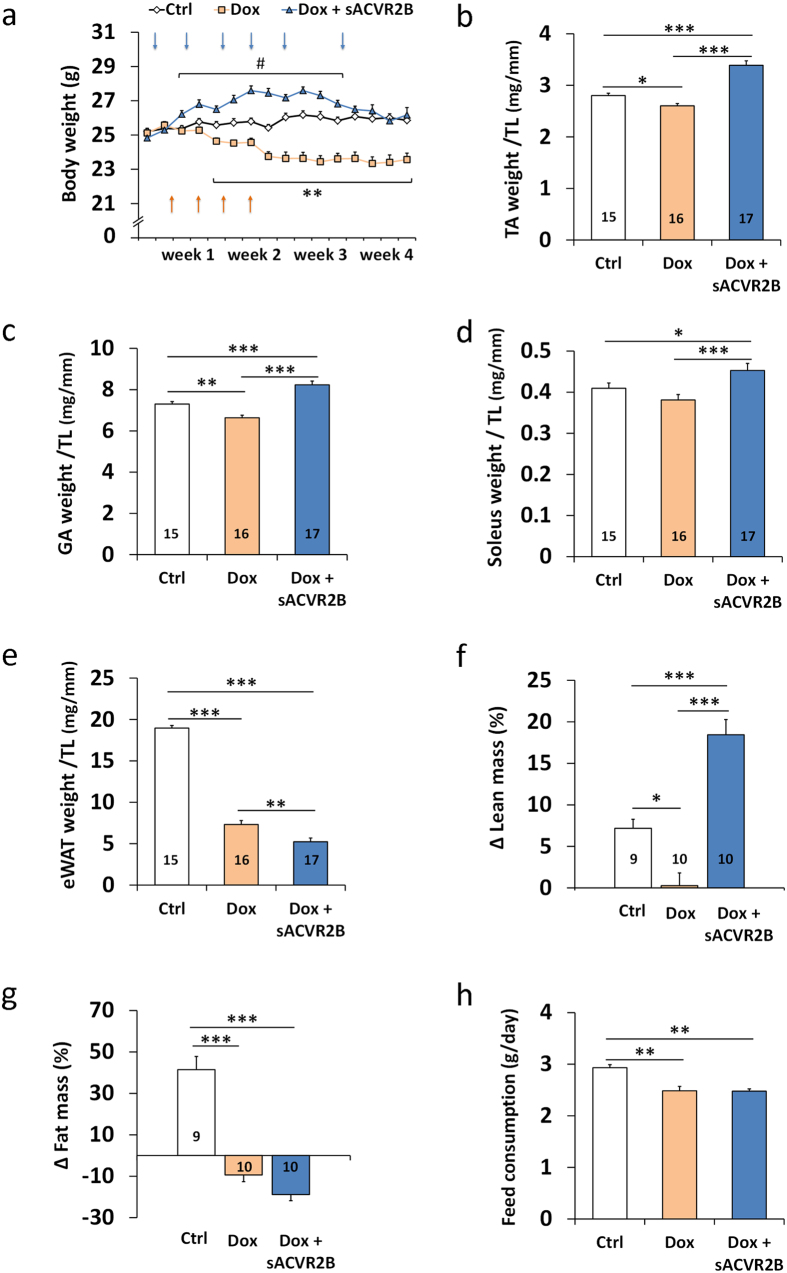
Doxorubicin administration resulted in decreased body and muscle weights that were restored by sACVR2B-Fc treatment. (**a**) Body weights during the four-week experiment. Arrows indicate the timing of doxorubicin (orange) and sACVR2B-Fc (blue) injections. Repeated measures ANOVA revealed time x group interaction effect (*P* < 0.001). Tissue weights of TA (**b**), gastrocnemius (GA) (**c**) and soleus (**d**) muscles and epididymal fat pads (**e**) relative to tibial length (Supplementary Fig. S1e). Percentage changes in lean (**f**) and fat (**g**) mass analysed with DXA. (**h**) Average feed consumption during and after the treatment with doxorubicin and sACVR2B-Fc. Average feed consumption per mouse was calculated from the pooled feed intake of the whole cage (2–3 mice/cage; N = 3–4 cages/group). N sizes are depicted in the bar graphs. Data are presented as mean ± SEM. In Fig. a: ^#^*P* < 0.05 compared to Ctrl; **P < 0.01 compared to Ctrl and Dox + sACVR2B (Bonferroni). In Figs. b–h: *P < 0.05; **P < 0.01; ***P < 0.001 (Bonferroni).

In the first experiments, mice were given a total cumulative dose of 24 mg/kg of doxorubicin (comparable to clinical dose^5^,^26^), or PBS, during the first two weeks of the experiment. In the four-week experiment, doxorubicin was not administered during the latter two weeks and thus the results represent more chronic effects of doxorubicin treatment. This doxorubicin administration resulted in marked decrease in body weight (Fig. 1a). The weight loss was most dramatic during the second week of doxorubicin administration and the reduced body weight was sustained after the cessation of doxorubicin administration (Fig. 1a). This was accompanied by a significant reduction in tissue masses of tibialis anterior (TA) and gastrocnemius muscles and epididymal fat pads determined upon euthanasia (Fig. 1b–e and Supplementary Fig. S1a–d). These findings were substantiated by dual-energy X-ray absorptiometry (DXA) analysis, which showed a significant decrease in lean mass and fat mass (Fig. 1f,g). sACVR2B-Fc treatment fully prevented the doxorubicin-induced decrease in body mass, lean mass and skeletal muscle weights and could even induce hypertrophy when compared to untreated healthy mice. However, sACVR2B-Fc treatment was unable to prevent the decrease in fat mass, which was even slightly exacerbated. Doxorubicin treated mice ate significantly less compared with the vehicle treated counterparts. sACVR2B-Fc treatment did not have any additional effect on feed consumption (Fig. 1h).

**Figure 2:**
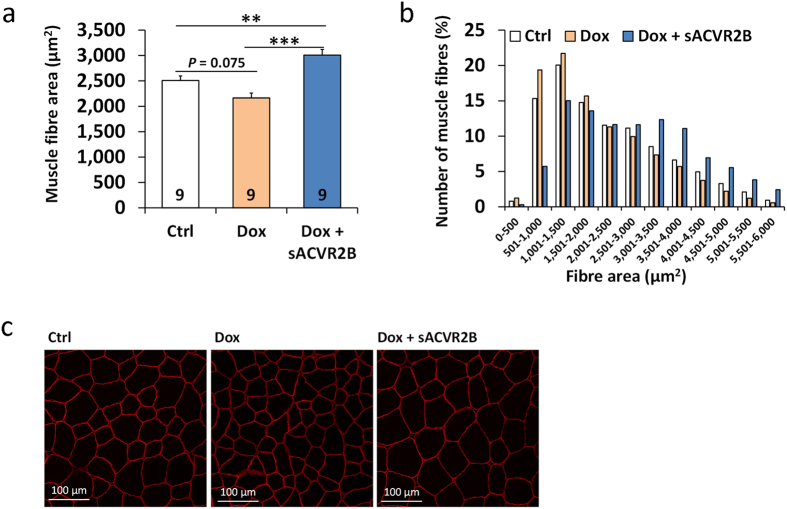
sACVR2B-Fc administration increased muscle fibre cross-sectional area in doxorubicin-treated mice. Average fibre CSA (**a**) and fibre size distribution (**b**) of the TA muscle at the end of the four-week experiment. (**c**) Representative immunofluorescence images of dystrophin-stained muscle cryosections. Data are presented as mean ± SEM. **P < 0.01; ***P < 0.001 (Bonferroni).

In line with skeletal muscle weights, doxorubicin treated mice tended (*P* = 0.075) to have decreased average muscle fibre cross-sectional area (CSA) in TA muscle (Fig. 2a,c) and fibre frequency curve shifted towards smaller fibres (Fig. 2b). This decrement was completely prevented by sACVR2B-Fc administration which resulted in significantly increased fibre size compared with both controls and doxorubicin treated mice (Fig. 2a–c).

### Blocking of the muscle loss prevents decrease in bone quality

**Figure 3:**
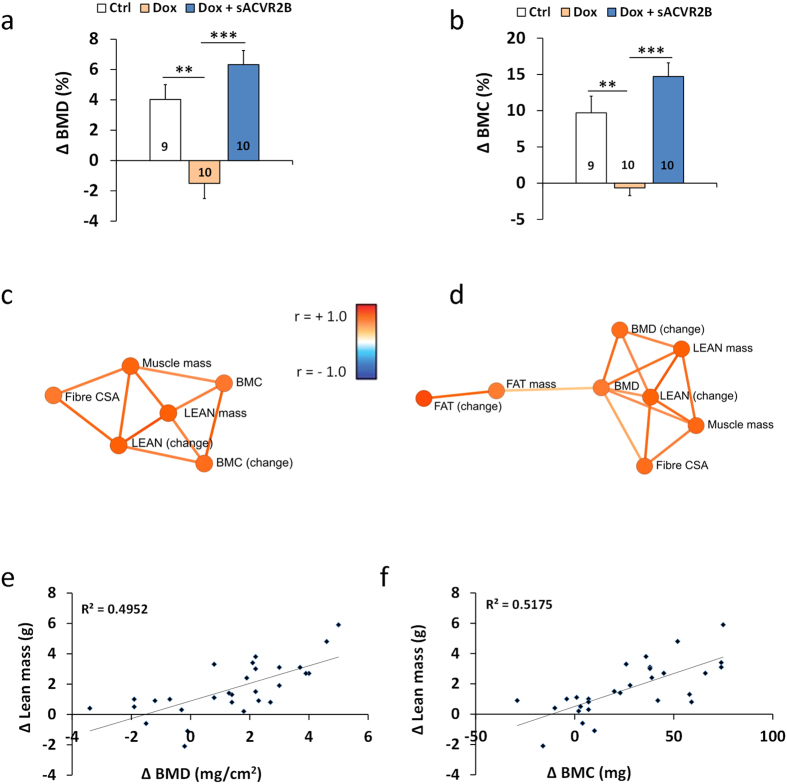
sACVR2B-Fc treatment improved bone quality in doxorubicin treated mice. Changes in bone mineral density (**a**) and content (**b**) in the four-week experiment. (**c**) Associations between muscle and lean mass and bone parameters. (**d**) Associations between muscle size, fat mass and BMD. Correlations between change in lean mass and change in BMD (**e**, r = 0.70, *P* < 0.001) and BMC (**f**, r = 0.72, *P* < 0.001). Data are presented as mean ± SEM. **P < 0.01; ***P < 0.001 (Bonferroni).

To further investigate the potential positive effects of maintaining muscle mass during chemotherapy, bones were analysed as a downstream outcome of the treatments. DXA-analysis showed a decrement in bone mineral density (BMD) and in bone mineral content (BMC) in doxorubicin-treated mice when compared to controls, whereas sACVR2B-Fc prevented these adverse effects (Fig. 3a,b). Our association analysis using a computationally determined network^27^ showed that BMD and BMC at the end and the change in BMD and BMC are highly correlated to changes in lean mass as well as the end-measures of muscle mass and muscle fibre CSA (Fig. 3c–f).

### Doxorubicin administration resulted in chronic impairment of treadmill running capacity

**Figure 4:**
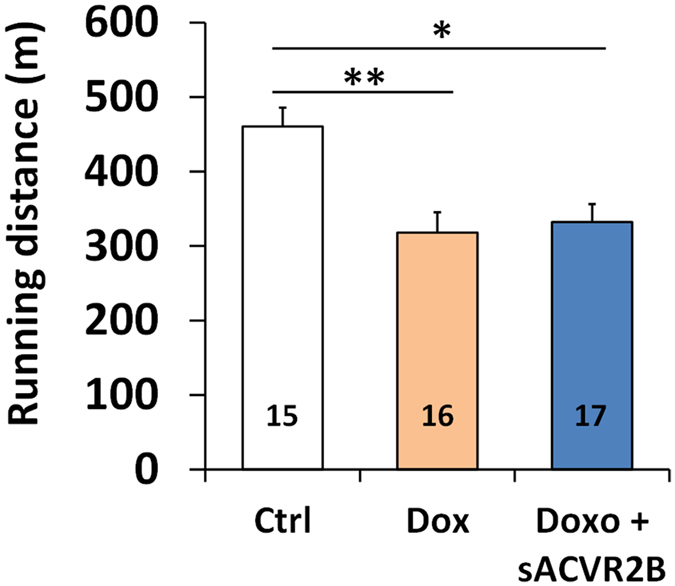
Doxorubicin-treated mice had significantly impaired running capacity with no effect of sACVR2B-Fc. Distance covered in an incremental treadmill running test until exhaustion. Data are presented as mean ± SEM. *P < 0.05; **P < 0.01 (Bonferroni).

Doxorubicin treated mice had significantly impaired maximal running performance in an incremental treadmill running test (Fig. 4). sACVR2B-Fc treatment did not have any effect on running performance.

### Doxorubicin induces muscle atrophy with only small alterations in proteolytic pathways

To investigate the factors that might contribute to doxorubicin-induced muscle atrophy and its reversal by sACVR2B-Fc, an acute experiment was conducted. Non-tumour bearing mice received a single intraperitoneal injection of doxorubicin (15 mg/kg in PBS) and were euthanized 20 hours post injection. Half of the doxorubicin treated mice were administered with sACVR2B-Fc (10 mg/kg in PBS) 48 hours before doxorubicin administration, as it has previously been reported that sACVR2B-Fc increases muscle protein synthesis 48 hours after its administration^19^. Microarray analysis was conducted from TA muscle to study gene expression responses to the treatments. To find out whether doxorubicin-induced muscle atrophy was due to increased protein catabolism, a common atrogene signature for genes that has previously been shown to be commonly down- or upregulated in fasting, cancer cachexia, renal failure, diabetes, and in loss of contractile activity^28^ was investigated (listed in Table 1). There were no systematic changes by doxorubicin or by sACVR2B-Fc on these transcripts (Table 1). There was however a minor trend that the atrogenes tended to follow the same trend in doxorubicin-administered mice as earlier^28^, although with a much smaller magnitude than with the other muscle wasting situations. Therefore, a gene set enrichment analysis (GSEA), able to detect small changes in several genes^29^, was conducted. Indeed, a custom-made gene set for these atrogenes showed a small increased enrichment (normalized enrichment score (NES) 1.44, FDR = 0.05) in doxorubicin administered mice when compared to vehicle treated control mice (Supplementary Fig. S2a) and this was blocked by sACVR2B-Fc administration (Supplementary Fig. S2b). GSEA analysis also revealed a small, but significant increase due to doxorubicin in the proteasome pathway (FDR = 0.041, Supplementary Fig. S2c,d) and also a trend in the caspase cascade (NES 1.75, FDR = 0.051) without any effect of sACVR2B-Fc. Of the individual atrogenes, *FOXO1* was the only one that was significantly (adjusted *P* < 0.05) induced by doxorubicin (Table 1, Supplementary Fig. S2a). Thus, FoxO1 protein level and its phosphorylation were analysed. Similarly as in the microarray analysis, total FoxO1 protein expression was increased significantly (*P* < 0.05) by doxorubicin, whereas phosphorylated FoxO1 remained at similar level between the groups (Supplementary Fig. S3a,e). In addition, mRNA expression of a well-known atrogene, MuRF1, was confirmed with qPCR, which, in accordance with the microarray result, showed no effect of doxorubicin treatment (Supplementary Fig. S3b). However, sACVR2B-Fc decreased MuRF1 mRNA (Supplementary Fig. S3b).

**Figure 5:**
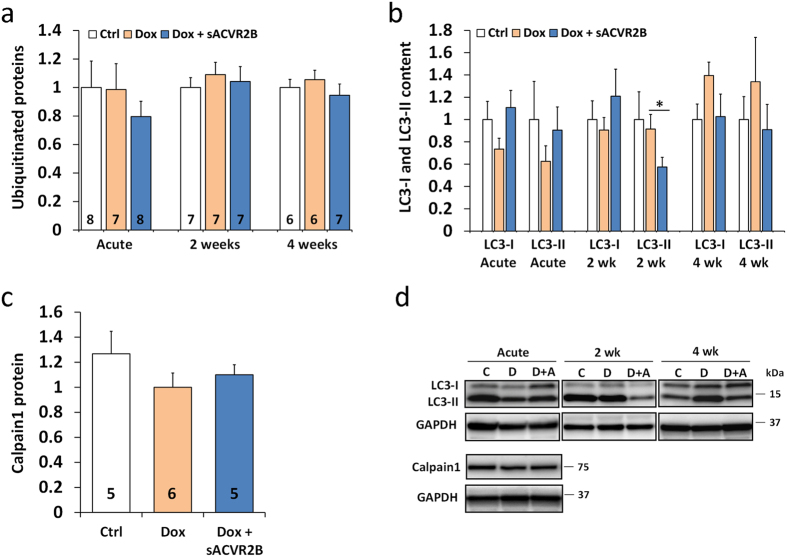
Doxorubicin administration did not cause marked alterations in the markers of ubituitin-proteasome system, autophagy or calpain1 content. Time-course of protein ubiquitination (**a**) and LC3-I and -II content (N = 5–9/group) (**b**) relative to Ctrl and calpain1 content at 20 h relative to Dox (**c**) in TA muscles. (**d**) Representative blots of LC3 and calpain1. Data are presented as mean ± SEM. *P < 0.05 (Mann-Whitney U). C = Ctrl; D = Dox; D+A = Dox+sACVR2B.

To study protein degradation pathways further, common markers of ubiquitin-proteasome system, autophagy and calpain content were analysed by western blotting. Doxorubicin administration did not result in any acute or chronic alterations in ubiquitinated proteins, lipidated LC3, or calpain1 protein content (Fig. 5a–d). No changes were noticed acutely or at 4 weeks, but at two weeks, sACVR2B-Fc treatment resulted in decreased lipidated LC3 (Fig. 5b). As LC3 lipidation alone is not sufficient marker for autophagy^30^, we also checked gene expression changes related to autophagy. Supporting the results above, autophagy gene set (KEGG) was not regulated by doxorubicin (FDR = 0.48, Supplementary Fig. S4a). Also after adjustment, the only significantly increased genes in this pathway were slightly increased Ulk1 (1.41 fold, *P* = 0.02) and Becn1 (1.31 fold, *P* = 0.01) in doxorubicin-administered mice, without marked effects of sACVR2B-Fc (Supplementary Fig. S4a,b). Doxorubicin has previously been shown to increase apoptosis, also in skeletal muscle^31^. Indeed, GSEA analysis revealed small increase in the gene set of apoptosis (NES 1.9, FDR = 0.01) without any effect of sACVR2B-Fc (Supplementary Fig. S4c,d).

To examine the possible delayed effects on muscle regeneration, TA muscles from mice from the four-week experiment were analysed for centrally nucleated fibres. Doxorubicin administration did not result in markedly and consistently increased number of centrally nucleated fibres (data not shown).

### Doxorubicin administration results in blunted skeletal muscle protein synthesis which is restored by sACVR2B-Fc treatment

**Figure 6:**
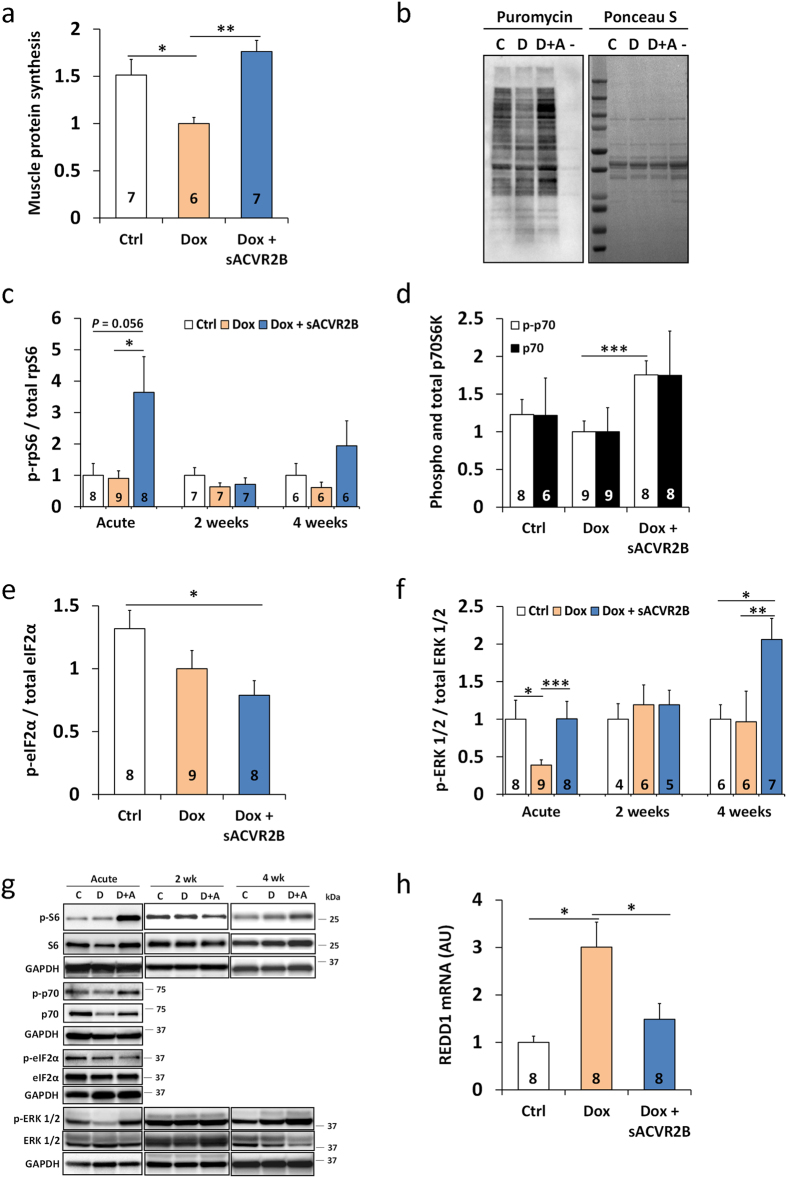
Doxorubicin administration resulted in decreased muscle protein synthesis that was restored by sACVR2B-Fc. (**a**) Muscle protein synthesis relative to Dox analysed with puromycin incorporation method and (**b**) representative blot (left) with Ponceau S staining (right). (− = negative control for puromycin). (**c**) Time-course of rpS6 phosphorylation at Ser240/244 relative to Ctrl in TA muscle. p70S6K (Thr389) (**d**) and eIF2α (Ser51) (**e**) phosphorylation response 20 hours after a single dose of doxorubicin relative to Dox. (**f**) Time-course of ERK 1/2 phosphorylation at Thr202/Tyr204 relative to Dox in TA muscle. (**g**) Representative blots of rpS6, p70S6K, eIF2α and ERK 1/2. (**h**) REDD1 mRNA expression normalized to 36b4 expression relative to Ctrl in TA muscle 20 hours after a single dose of doxorubicin. Data are presented as mean ± SEM. *P < 0.05; **P < 0.01; ***P < 0.001 (Bonferroni (**a**;**h**), Mann-Whitney U -test (**c**–**f**)).

As only small effects were seen in the expression of negative regulators of muscle size, the effects of doxorubicin and sACVR2B-Fc administration on positive regulators of muscle mass and protein synthesis were investigated. To study protein synthesis, a previously published method of surface sensing of translation (SUnSET)^32^,^33^ was applied as earlier in our laboratory^19^. This analysis revealed that muscle protein synthesis was significantly blunted 20 hours after doxorubicin administration compared with the controls (Fig. 6a,b). This decrease in protein synthesis was completely inhibited in mice treated with sACVR2B-Fc 48 hours prior to the exposure to doxorubicin (Fig. 6a,b). This was accompanied by increased mTORC1 signalling illustrated by elevated phosphorylation of its downstream targets rpS6 and p70S6K1 in sACVR2B-Fc treated mice acutely, but less so in the later time-points (Fig. 6c,d,g). However, at all the time-points investigated, no change in the activation of mTORC1 signalling was observed by doxorubicin, which suggests that doxorubicin and sACVR2B-Fc are affecting different pathways regulating muscle mass. Additionally, no changes due to the treatments were observed in the phosphorylation of Akt (at Ser473) or the phosphorylation of 4EBP1 (at Thr37/46) (Supplementary Fig. S3c–e).

Increased phosphorylation of eIF2α on Ser51 inhibits translation initiation and it has been associated with decreased muscle protein synthesis^34^ and cachexia^14^. However, the western immunoblot analysis did not show any significant doxorubicin-induced changes in eIF2α phosphorylation (Fig. 6e,g). Interestingly, sACVR2B-Fc decreased the phosphorylation of eIF2α compared to control (Fig. 6e,g). Another pathway regulating muscle size, partially parallel to mTORC1 signalling, is MAPK signalling. The phosphorylation of ERK 1/2 was acutely downregulated in doxorubicin treated mice, while sACVR2B-Fc treatment prevented this decrease (Fig. 6f,g). At two and four weeks, the phosphorylation level of ERK 1/2 was similar between doxorubicin only treated mice and control mice.

### REDD1 is highly upregulated in doxorubicin-induced muscle loss

To unravel the factors potentially underlying doxorubicin-induced decrease in protein synthesis and muscle size, microarray data was analysed for genes most induced by doxorubicin known to regulate muscle protein synthesis and size. Doxorubicin treated mice showed a significant 2-fold (adjusted *P* = 0.02) increase in mRNA expression of *REDD1*, a protein previously connected to muscle wasting^14^,^35^. This increase was confirmed by qPCR, which showed a 3-fold increase in *REDD1* expression in doxorubicin treated mice (Fig. 6h). The effect of doxorubicin on *REDD1* expression was partially blocked by sACVR2B-Fc treatment.

### Skeletal muscle mitochondrial function and content are not chronically altered in response to doxorubicin administration

**Figure 7:**
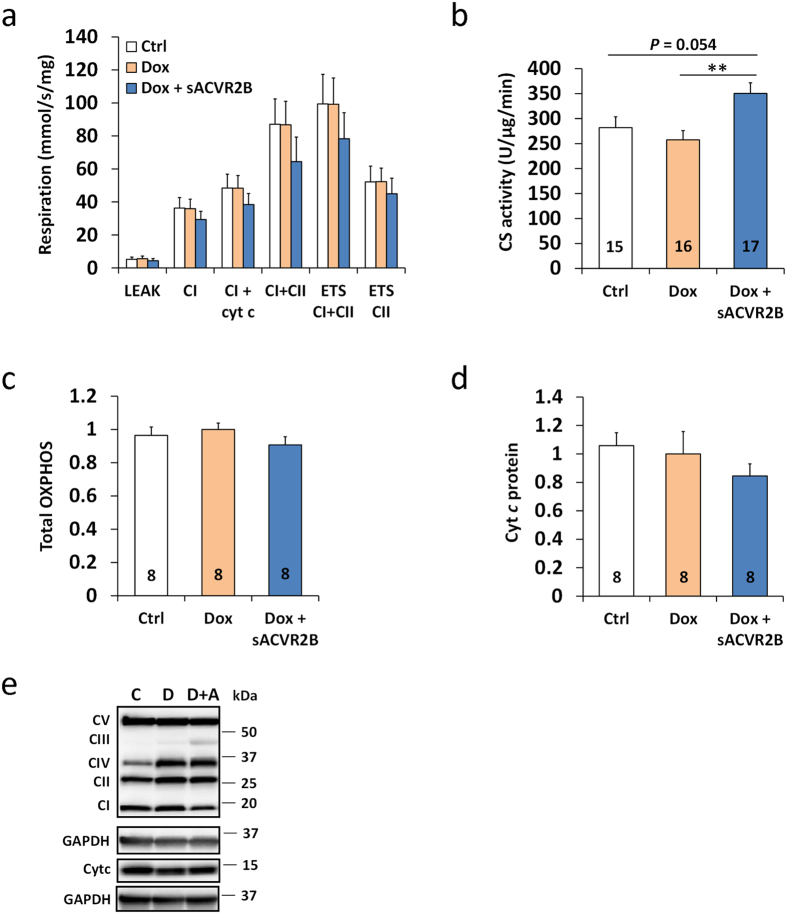
Doxorubicin administration did not affect mitochondrial function or markers of mitochondrial content in skeletal muscle. (**a**) Mitochondrial respiration in homogenized TA muscle with carbohydrate substrates (N = 7–8/group). Cyt c = cytochrome *c*; CI/II = complex I/II; ETS = electron transfer system. (**b**) Citrate synthase (CS) activity measured from TA muscle. Quantification of total content of mitochondrial respiratory chain subunits (**c**) and cytochrome *c* (Cyt *c*) relative to Dox (**d**) and representative blots (**e**). Data are presented as mean ± SEM. **P < 0.01 (Bonferroni (**a**–**b**), Mann-Whitney U (**c**–**d**)).

To explore potential factors explaining impaired running capacity of the doxorubicin treated mice, skeletal muscle mitochondrial function was analysed with OROBOROS Oxygraph-2k high-resolution respirometer. At the four-week time-point, two weeks after cessation of doxorubicin administration, the analysis showed no differences in mitochondrial respiratory function of TA muscle between the three groups (Fig. 7a). This occurred independent of whether the results were presented as oxygen flux per wet weight of tissue or normalized to total TA weight or to an index of mitochondrial content. To study skeletal muscle mitochondrial function and content further, citrate synthase activity and expression of several mitochondrial proteins were analysed. Doxorubicin did not seem to alter citrate synthase activity, while increased activity was seen in mice treated with sACVR2B-Fc (Fig. 7b). Similarly as mitochondrial function, the total content of mitochondrial respiratory chain subunits (total OXPHOS) and cytochrome *c* (cyt *c*) protein remained unchanged in TA muscle irrespective of the treatment (Fig. 7c–e). However, an ion channel protein between cytosol and mitochondrial matrix, porin/VDAC1^36^, was significantly elevated in sACVR2B-Fc treated mice (Supplementary Fig. S5a,h). Of individual OXPHOS proteins, mitochondrial respiratory chain CI-NDUFB8 and CV-ATP5A were decreased in sACVR2B-Fc treated mice compared with doxorubicin only treated mice (Supplementary Fig. S5b–f). No differences were detected in the PGC-1α protein (Supplementary Fig. S5g,h) or different PGC-1α isoforms by either doxorubicin alone or combined with sACVR2B-Fc (Supplementary Fig. S6a–d).

### Skeletal muscle oxygen carrying capacity, but not capillary density, is altered in response to doxorubicin administration

To study if blood oxygen carrying capacity was altered due to the treatments, haematological parameters were investigated at two and four weeks. Doxorubicin administration independently of sACVR2B-Fc administration resulted in decline in blood haemoglobin and haematocrit at two weeks (immediately after treatment), but this effect disappeared at four weeks (Supplementary Fig. S7a–d).

**Figure 8:**
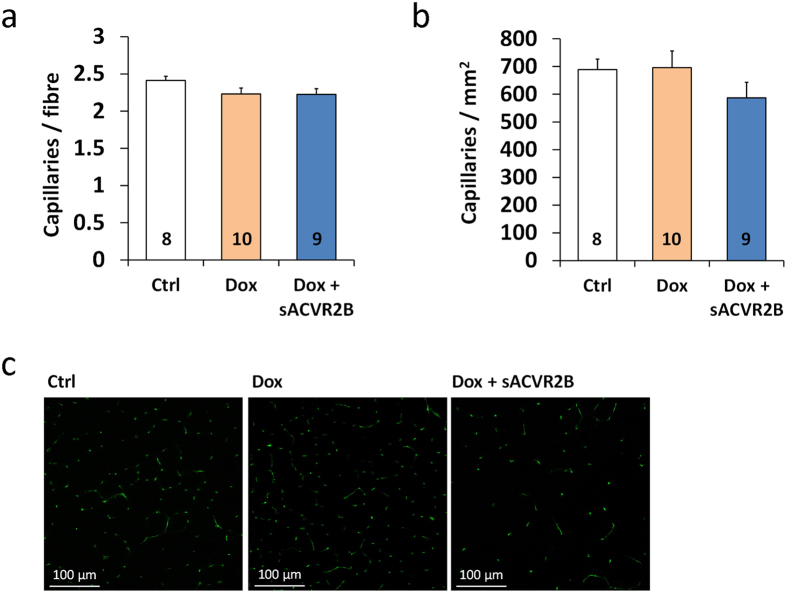
Doxorubicin administration did not affect skeletal muscle capillary density. Quantification of the capillary-to-fibre ratio (**a**) and the number of capillaries per muscle area (**b**) in TA muscle. (**c**) Representative immunofluorescence images of CD31/PECAM-1 staining for capillaries. Notice that the representative capillary images are from exactly the same location as the dystrophin staining in Fig. 2c. Data are presented as mean ± SEM. *P < 0.05 (Bonferroni).

As no major differences were found in skeletal muscle mitochondrial function or content, capillary count was analysed from TA. There were no significant effects of doxorubicin or sACVR2B-Fc treatment on capillary-to-fibre ratio or capillary density (Fig. 8a–c).

### sACVR2B administration did not have any effect on tumour growth or on the antineoplastic effect of doxorubicin

To investigate whether the sACVR2B-Fc treatment has effect on the antineoplastic effect of chemotherapy or tumour growth, a two-week tumour experiment using the Lewis lung carcinoma (LLC) model was conducted. LLC-cancer did not induce muscle atrophy yet at this time-point (Supplementary Fig. S8a–d). Similarly as with doxorubicin treated non-tumour bearing mice, doxorubicin treatment seemed to be associated with increased loss of lean and fat mass also in tumour bearing mice (Supplementary Fig. S8c–e,g,h) despite relatively low cumulative dose (12 mg/kg) of doxorubicin used. sACVR2B-Fc increased skeletal muscle and lean mass similarly in tumour bearing mice irrespective of doxorubicin administration (Supplementary Fig. S8c,d,g). Interestingly, in contrast to the results from doxorubicin experiments in non-tumour bearing mice, in this setting, sACVR2B-Fc treatment also protected from the excessive loss of epididymal fat by doxorubicin, but not from the loss of total fat mass (DXA) (Supplementary Fig. S8e,h). The tumours of the sACVR2B-Fc treated mice had similar response to doxorubicin treatment compared to mice not treated with sACVR2B (Supplementary Fig. S8f). These results suggest that inhibiting ACVR2B ligands can decrease the cachectic effects of chemotherapy without adversely affecting tumour growth or compromising the antineoplastic effect of chemotherapy on the tumour.

## Discussion

Cancer-therapy aiming to treat malignancies can be associated with toxicities in various tissues, such as skeletal muscle, and also with shorter life span^2^,^3^. In the present study we show that prevention of doxorubicin chemotherapy-induced muscle atrophy can be achieved by preventing decreased muscle protein synthesis using a blocker for ACVR2B ligands without negative side-effects on aerobic capacity or tumour growth.

Doxorubicin administration resulted in marked decrease in body weight, comprised of loss of both lean and fat mass. Skeletal muscle atrophy was observed as a decrease in muscle masses and in TA fibre size. These results are consistent with previous studies^10^,^11^,^37^. The reduced fat mass and muscle size by doxorubicin might be at least partially explained by reduced feed intake. However, as doxorubicin has been shown to accumulate in skeletal muscle tissue^38^, it is possible that doxorubicin also had a direct effect on skeletal muscle. Indeed, doxorubicin accumulation was detected in skeletal muscles, and it acutely led to a typical p53 and DNA-damage response (unpublished observations) in line with previous reports in cardiac muscle^5^,^39^. Importantly, treatment of muscle atrophy by blocking ACVR2B ligands was accomplished without any adverse effects on tumour, as shown earlier with several other tumour models^20^,^21^, or on the effect of doxorubicin on tumour.

Muscle atrophy is a result of a situation in which the rate of protein synthesis is, over a period of time, repressed relative to that of degradation. Protein synthesis responses in muscle atrophy have been overall less investigated than protein degradation pathways^13^,^14^. The present results show, for the first time, that doxorubicin administration acutely results in blunted protein synthesis in skeletal muscle. Previous studies have reported either repressed^40^ or unchanged^41^ protein synthesis in cardiac muscle or cardiomyocytes after acute doxorubicin administration. Importantly, the decreased muscle protein synthesis by doxorubicin was completely prevented by sACVR2B-Fc administration. The increased protein synthesis by sACVR2B-Fc is likely due to increased mTORC1 signalling manifested by increased phosphorylation of p70S6K and rpS6. These markers of mTORC1 signalling also positively correlated with protein synthesis (Supplementary Fig. S9a). This is consistent with previous results from our laboratory, which showed that sACVR2B-Fc treatment in healthy wildtype mice increased muscle size, protein synthesis and mTORC1 signalling^19^. However, doxorubicin did not seem to affect this pathway at least at 20 h time-point as phosphorylation levels of p70S6K and rpS6 were similar to control group. This does not exclude the possibility that decrease in mTORC1 signalling would have preceded the decreased protein synthesis.

In the present study, no major changes were observed in protein degradation markers such as ubiquitinated proteins, atrogene expression^28^, or markers of autophagy. However, the slight enrichment of the atrogene, proteasome and apoptosis gene sets in response to doxorubicin administration might indicate at least a small increase in protein degradation and apoptosis. The previous available evidence on protein degradation and apoptosis pathways suggests activated calpain-caspase-3-apoptosis pathway^9^,^11^,^42^, and increased markers of ubiquitin–proteasome system^9^, and autophagy^43^ as contributors to doxorubicin-induced muscle atrophy, but the evidence is inconsistent^31^,^44^. The reason why we observed only very small upregulation in the protein degradation and apoptosis pathways can be speculated to be the dosage of doxorubicin used in our study, i.e. 24 mg/kg cumulative dose or 15 mg/kg single dose in the acute experiment. The 15–24 mg/kg in mice is equivalent to ~45–72 mg/m^2^ in humans and thus very close to the clinical doses used in cancers (30–90 mg/m^2^)^5^. These doses are relatively small compared to the ones typically used in the rat studies, i.e. 20 mg/kg in rats^11^,^37^,^42^ that can be estimated to be equivalent to ~40 mg/kg in mice and ~120 mg/m^2^ in humans^26^. Nevertheless, although the mechanisms behind doxorubicin-induced muscle atrophy can be dose-dependent^9^, the blockade of decreased protein synthesis without major alterations in the protein degradation, apoptosis or autophagy pathways seems to be the mechanism by which sACVR2B-Fc prevents doxorubicin-induced muscle loss. However, part of the increased protein synthesis may also be due to larger *de novo* protein synthesis, as we have shown earlier that sACVR2B-Fc increases muscle protein synthesis also in healthy mice^19^. Moreover, a decrease in the ubiquitin ligase MuRF1 mRNA by sACVR2B-Fc was noticed supporting previous studies by Rahimov *et al*. in wildtype mice and Zhou *et al*. in cachectic mice^20^,^25^. This shows a potential for sACVR2B-Fc in preventing atrophy also in situations where protein degradation pathways are more strongly activated.

A transcriptome analysis was conducted to investigate if genes previously shown to have a role in muscle atrophy were regulated by doxorubicin. Interestingly, REDD1, a DNA damage marker protein that has previously been connected to muscle wasting and decreased protein synthesis^14^ also in other models, e.g. streptozotocin-induced experimental type 1 diabetes^35^, was one of the most highly upregulated genes. A negative correlation between REDD1 expression and protein synthesis was observed (Supplementary Fig. S9b). This suggests that doxorubicin-induced REDD1 expression could, in part, contribute to decreased protein synthesis, but more mechanistic evidence is needed to verify this connection. Another candidate associated with the regulation of muscle size is MAPK-signalling^45^,^46^. Doxorubicin administration resulted in marked decrease in ERK 1/2 phosphorylation. Similar decrease in ERK phosphorylation has been previously reported five days after doxorubicin dose identical to our acute experiment (15 mg/kg) in mouse skeletal muscle^31^. The physiological importance of altered ERK 1/2 MAPK-signalling is unknown, and may be dependent on time-point and context^19^,^45^,^46^,^47^. Interestingly, in our setting, sACVR2B-Fc administration restored ERK 1/2 phosphorylation to the level of control mice. We have shown earlier that sACVR2B-Fc treatment decreased the phosphorylation of ERK 1/2 at early, but not at later time-points in healthy mice^19^. This shows that, in addition to the many other pathways, such as mTORC1 signalling, the regulation of MAPK signalling by blocking ACVR2B receptor ligands can also be dependent on timing and context. More research is needed to determine the consequences and importance of the doxorubicin-induced decrease in ERK 1/2 phosphorylation and prevention of this response by blocking sACVR2B ligands.

Several studies have investigated the effects of exercise training on the effects of doxorubicin. Majority^42^,^43^,^48^, but not all^49^ of these studies suggest that exercise training protects against the adverse effects of doxorubicin on cardiac and skeletal muscle without compromising^50^ or even enhancing its antitumor efficacy^49^. On the other hand, maximal aerobic capacity on a whole-body level has been associated with longevity and health^51^. Muscle weakness and fatigue as well as skeletal muscle contractile dysfunction have been reported after exposure to doxorubicin in patients^4^ and in animal models *ex vivo*^4^,^8^,^12^. However, to the knowledge of the authors, the present study is the first one to show direct negative effects of doxorubicin administration on maximal aerobic running capacity *in vivo*. As this effect was observed two weeks after the cessation of doxorubicin administration, it seems that the impairment in aerobic capacity is sustained.

Impaired running performance by doxorubicin could have been expected to be accompanied by decreased mitochondrial respiratory capacity, but this was not the case in the present study. According to the previous literature, doxorubicin can cause impaired mitochondrial function in cardiac^52^,^53^ and in skeletal muscle^10^,^37^,^53^. However, the evidence concerning skeletal muscle is inconsistent: not all studies report significant impairments in the markers of mitochondrial function in response to doxorubicin treatment^52^. In addition, Gouspillou and colleagues^10^ did not observe any significant or sustained impairment in skeletal muscle mitochondrial respiratory function after two cycles of doxorubicin treatment (cumulative dose 20 mg/kg) in mice. However, four cycles of treatment (cumulative dose 40 mg/kg) resulted in impaired mitochondrial respiration that was sustained over a 12-week period after the last cycle^10^. In that study mice were also treated with dexamethasone, so the contribution of each drug to the effects cannot be confirmed^10^. Furthermore, impaired mitochondrial respiration has been reported 2–72 hours after a single high dose of doxorubicin (20 mg/kg) in rats^37^. According to the present and these previous studies, the dosage of doxorubicin used and the time-points investigated may play important roles in doxorubicin-induced alterations in skeletal muscle mitochondrial function.

As a novel finding of the present study, doxorubicin treatment did not alter muscle capillary density and thus, decreased capillarization cannot explain the impaired running capacity. Also limb muscle independent factors can lie behind the persistently impaired running performance. Systemic doxorubicin administration has previously been shown to cause weakness and contractile dysfunction in diaphragm^11^,^12^, the principal respiratory muscle. Doxorubicin-induced cardiotoxicity can also have an effect on whole body exercise capacity. Interestingly, sACVR2B-Fc could not prevent doxorubicin-induced cardiac atrophy (data not shown). This was, however, achieved by vascular endothelial growth factor-B (VEGF-B^54^) gene therapy in the same experimental setting (Räsänen *et al*. unpublished observations). The present study also showed that doxorubicin administration can reduce blood oxygen carrying capacity consistent with earlier reports^55^. However, this did not persist anymore two weeks after the cessation of doxorubicin administration. Thus, this effect probably does not play a major role in the persistently impaired running performance. It is likely that multiple factors, rather than just one, contribute to impaired exercise capacity by doxorubicin.

Contrary to previous results in wild type and dystrophic mice^18^,^23^, systemic sACVR2B-Fc administration did not cause any further impairment in running performance or mitochondrial content or function. Some more specific effects were, however, noticed. Unlike in wildtype or mice with muscular dystrophy^18^,^23^, citrate synthase activity and the content of a mitochondrial channel protein porin/VDAC1^36^ were increased in doxorubicin treated mice administered with sACVR2B-Fc compared with doxorubicin alone. However, sACVR2B-Fc administration decreased mitochondrial respiratory chain subunit proteins CI-NDUFB8 and CV-ATP5A. This suggests that blocking ACVR2B ligands may have specific effects on mitochondrial proteins even though overall mitochondrial capacity or function may be unaltered. Previously, sACVR2B-Fc administration has decreased electron transport chain and oxidative phosphorylation gene sets in mdx mice^22^. As no effects on mitochondrial function or further decrease in running capacity were detected, these specific effects of blocking ACVR2B ligands have probably only minor physiological significance. Our laboratory has previously published evidence of interaction effects of exercise and sACVR2B-Fc in muscles^18^,^22^. Future studies should investigate the effects of blocking activin receptor ligands also in active mice.

Musculoskeletal system plays an important role in e.g. enabling locomotion. Bone can serve as an ion reserve to maintain serum ion concentrations of e.g. calcium and magnesium. Mechanical and molecular interaction between muscles and bone has got lots of attention during the last few years^56^. Many studies have shown that improving muscle size and strength can improve bone quantity or quality^57^. For instance, blocking myostatin/activins has improved bone mass, quality and strength^58^. On the other hand, bone parameters have been reported to be decreased in rodents after doxorubicin administration^59^. The present study showed that the decrease in BMD and BMC by doxorubicin was prevented by sACVR2B-Fc and that the bone results are strongly related to muscle mass. Therefore, it is speculated that changes in bone adaptation were secondary to muscle showing the importance of muscle *per se* on certain markers of health. However, the possible existence of other direct or indirect effects of sACVR2B-Fc on bone cannot be excluded^58^.

In conclusion, unlike in many other cachexia-inducing diseases, our findings show that doxorubicin chemotherapy induces skeletal muscle atrophy without markedly increasing typical atrogenes or protein degradation pathways. In contrast, muscle atrophy induced by doxorubicin is probably mainly mediated by decreased protein synthesis, and this effect is prevented by blocking ACVR2B signalling. In addition, the current results suggest that blocking ACVR2B signalling may be a promising strategy to counteract chemotherapy-induced muscle and bone loss without further damage to skeletal muscle oxidative capacity or mitochondria or the actual treatment of malignancies.

## Methods

### Animals

C57BL/6J male mice (Envigo), aged 9–10 weeks, were used in all experiments. Mice were maintained under standard conditions (temperature 22 °C, 12:12 h light/dark cycle) with free access to food and water. The protocols were approved by the National Animal Experiment Board, and all the experiments were carried out in accordance with the guidelines of that committee.

### Experimental design

Five experiments were conducted: 1–2) two four-week experiments, 3) a two-week experiment, 4) an acute experiment, and 5) a tumour experiment. In experiments 1–4, the mice were randomly assigned into one of three groups: 1) vehicle (PBS) treated controls (Ctrl), 2) doxorubicin hydrochloride treated mice (Dox), and 3) doxorubicin treated mice administered intraperitoneally with sACVR2B-Fc (Dox + sACVR2B). In the tumour experiment (exp 5), the mice were randomized into five groups: 1) healthy controls (Ctrl), 2) LLC-tumour bearing mice (LLC + PBS), 3) LLC- mice treated with doxorubicin (LLC + Dox), 4) LLC-mice treated with sACVR2B-Fc (LLC + sACVR2B), and 5) LLC-mice treated with doxorubicin and sACVR2B-Fc (LLC + Dox + sACVR2B).

### Experimental treatments

In experiments 1–3, all doxorubicin administered mice received a total of four intraperitoneal injections of doxorubicin (6 mg/kg in PBS), administered every third day during the first two weeks of the experiment. Control mice were administered with an equal volume of PBS. In the four-week experiments (1 and 2), the mice were euthanized four weeks after the first and 19 days after the last doxorubicin injection. In the two-week experiment (3), the mice were euthanized two weeks after the first and 4 days after the last doxorubicin injection. In these experiments, sACVR2B-Fc (5 mg/kg in PBS) was administered intraperitoneally for half of the doxorubicin treated mice twice a week during the first two weeks of the experiment and once a week after that (in the 4-week experiments). sACVR2B-Fc administration was started before the first doxorubicin injection and the last dose was administered seven days before euthanasia.

In the acute experiment, doxorubicin treated mice received a single intraperitoneal injection of doxorubicin (15 mg/kg in PBS) and controls an equal volume of PBS. sACVR2B-Fc treated mice received a single intraperitoneal injection of sACVR2B-Fc (10 mg/kg in PBS) 48 hours before doxorubicin administration, a time-point when sACVR2B-Fc shows increased muscle protein synthesis^19^. The mice were euthanized 20 hours after doxorubicin/PBS administration.

In the tumour experiment, mice were subcutaneously inoculated with 0.5 × 10^6^ LLC cells (a kind gift from Dr. Alitalo) in 100 μl of PBS or with an equal volume of vehicle only (controls) into the right abdominal region. Doxorubicin was administered intraperitoneally twice during the experiment on the sixth and eleventh day after tumour inoculation (cumulative dose 12 mg/kg). sACVR2B-Fc (5 mg/kg in PBS) was administered intraperitoneally twice a week starting from third day after tumour inoculation and the last injection being two days before euthanasia. The mice were euthanized 14 days after tumour inoculation.

### Tissue collection

At the end of all experiments, the mice were anaesthetized and then euthanized by heart puncture followed by cervical dislocation. Hindlimb muscles TA, gastrocnemius and soleus as well as epididymal fat pads were immediately excised and weighed. The left TA was snap-frozen in liquid nitrogen and the right TA muscle was mounted in O.C.T. embedding medium (Tissue Tek) and snap-frozen in isopentane cooled with liquid nitrogen. All tissue weights were normalized to the length of the tibia (mm).

### sACVR2B-Fc production

The recombinant fusion protein was produced and purified *in house* as described earlier in detail^19^. Briefly, the ectodomain of human ACVR2B was fused with a human IgG1 Fc domain and the fusion protein was expressed in Chinese hamster ovary cells grown in a suspension culture. The protein is similar, but not identical to that originally generated by Lee and colleagues^17^.

### Tumour cell line and cell culture

LLC cells were originally purchased from American Type Culture Collection (Manassas, VA) and maintained in complete Dulbecco’s Modified Eagle’s Medium (DMEM) supplemented with 2 mmol/L L-glutamine, penicillin (100 U/mL), streptomycin (100 μg/mL), and 10% FBS.

### Dual-energy X-ray absorptiometry (DXA)

For the DXA analysis, mice were anaesthetized with a combination of ketamine and xylazine and imaged with Lunar PIXImus II densitometer (GE Healthcare). The images were analysed using standard procedures.

### Treadmill running protocol

The mice ran first at 9, 12 and 15 m/min for 5 minutes each, after which the velocity was increased by 2 m/min every 2 minutes until exhaustion. All mice were familiarized with treadmill running on a separate day prior to the test.

### Muscle protein synthesis: *in vivo* surface sensing of translation

Muscle protein synthesis was analysed using surface sensing of translation (SUnSET) method^32^,^33^ as earlier in our laboratory^19^. At exactly 25 min after puromycin (Calbiochem) administration, mice were euthanized by heart puncture followed by cervical dislocation. Left TA muscle was isolated, weighed and snap-frozen in liquid nitrogen exactly 30 minutes after puromycin administration.

### Mitochondrial function analysis

Skeletal muscle mitochondrial function was analysed with OROBOROS Oxygraph-2k high-resolution respirometer with similar procedures as earlier^54^. Briefly, a thin cross-section of 5–10 mg from the middle of the left TA muscle was removed and temporarily stored in Biops buffer. The sample was then homogenized with a shredder and carbohydrate SUIT protocol was used to analyse mitochondrial function as previously described^54^.

### RNA analysis

Total RNA was extracted from the TA muscle with TRIsure reagent (Bioline) and further purified with NucleoSpin^®^ RNA II columns. For qPCR RNA was reverse transcribed to cDNA using iScript^TM^ Advanced cDNA Synthesis Kit for RT-qPCR (Bio-Rad Laboratories) according to the manufacturer’s instructions. Real-time qPCR was performed according to standard procedures using iQ SYBR Supermix (Bio-Rad Laboratories) and CFX96 Real-Time PCR Detection System (Bio-Rad Laboratories). Data analysis was carried out by using efficiency corrected ΔΔCt method. More information on qPCR is given in Supplementary methods.

RNA samples of five mice from each group were analysed with Illumina Sentrix MouseRef-6 v2 Expression BeadChip containing 45281 transcripts (Illumina Inc.) by the Functional Genomics Unit at Biomedicum Helsinki, University of Helsinki, Finland. Before the analysis, sample RNA was analysed for integrity and quality with Agilent Bioanalyzer 2100. Raw data were normalized with quantile normalization and data quality was assessed using Chipster software (IT Center for Science, Espoo, Finland)^60^. The complete data set is publicly available in the NCBI Gene Expression Omnibus (http://www.ncbi.nlm.nih.gov/geo/; accession no. GSE77745). More detailed description is provided in Supplementary methods.

### Protein extraction and content

TA muscle samples were homogenized in ice-cold buffer with proper inhibitors and further treated as earlier^19^,^35^ with slight modifications. Total protein content was determined using the bicinchoninic acid (BCA) protein assay (Pierce, Thermo Scientific) with an automated KoneLab device (Thermo Scientific).

### Citrate synthase activity

Citrate synthase activity was measured from TA muscle homogenates using a kit (Sigma-Aldrich) with an automated KoneLab device (Thermo Scientific).

### Western blotting

Western immunoblot analyses were performed as previously reported^19^,^35^, with slight modifications in the quantification of ubiquitinated proteins and puromycin incorporation: In the case of the analysis of puromycin-incorporated proteins and ubiquitinated proteins, the intensity of the whole lane was quantified. Ponceau S staining and GAPDH were used as loading controls and all the protein level results were normalized to the mean of Ponceau S and GAPDH, except for the puromycin-incorporated proteins that were normalized only to Ponceau S. The antibodies used are listed in the supplementary methods online.

### Muscle immunohistochemistry

Cross-sections (10 μm) were cut from TA muscle with a cryomicrotome. To analyse muscle fibre cross-sectional area (CSA), the sarcolemmas were visualized using antibodies against Dystrophin (Abcam) with Alexa Fluor 555 secondary antibody (Molecular Probes). This was combined with PECAM-1/CD31 (BD Pharmingen) staining with Alexa Fluor 488 secondary antibody (Molecular Probes) to visualize capillaries and DAPI for the nuclei. The stained sections were imaged with a confocal microscope (Zeiss) and ZEN software. The mean fibre CSA was quantified from 1,065 ± 58 fibres representing both the deep and the superficial regions of the muscle. For the analysis of fibre size distribution, 550 fibres were randomly picked from each muscle sample. Muscle fibre CSA, capillary density (capillaries/mm^2^) and capillary-to-fibre ratios were analysed with ImageJ software (NIH).

### Statistical analyses

Values are presented as means ± SEM. The data from experiments 1 and 2 was pooled when there were no differences between the experiments. Data was checked for normality and differences between groups were analysed with general linear model ANOVA with Bonferroni post-hoc test, when appropriate. Western blot results were analysed with general linear model ANOVA with Bonferroni post-hoc test or with non-parametric Kruskal-Wallis test with Holm-Bonferroni corrected Mann-Whitney U as post-hoc when appropriate. Correlations were analysed using Pearson’s Product Moment Coefficient. Differences were considered statistically significant at *P *≤ 0.05. Statistical analyses were performed with IBM SPSS Statistics version 22 for Windows (SPSS, Chicago, IL).

## Additional Information

**How to cite this article**: Nissinen, T.A. *et al*. Systemic blockade of ACVR2B ligands prevents chemotherapy-induced muscle wasting by restoring muscle protein synthesis without affecting oxidative capacity or atrogenes. *Sci. Rep.* **6**, 32695; doi: 10.1038/srep32695 (2016).

## Supplementary Material

Supplementary Information

## Figures and Tables

**Table 1:** Atrogenes previously altered by systemic diseases in mice (fasting, tumor, uremia, diabetes mellitus) as well as disuse^28^.

Accession no.	Gene	Dox vs. Ctrl		Dox + sACVR2B vs. Dox	
		FC	*P* adj.	FC	*P* adj.
Protein degradation					
NM_001039048.2	MuRF1	1.01	0.979	0.72	0.322
NM_026346.1	Fbxo32	1.81	0.200	0.46	0.196
NM_009984.2	Ctsl	0.99	0.976	0.97	0.931
NM_011971.4	Psmb3	1.04	0.564	1.03	0.841
NM_026545.2	Psmd8	0.89	0.134	1.27	0.041*
NM_178616.2	Psmd11	1.02	0.859	0.95	0.882
NM_008945.2	Psmb4	1.07	0.439	0.97	0.884
XM_001479832.1	UBC	1.07	0.469	0.95	0.848
NM_021522.2	Usp14	1.06	0.797	1.09	0.898
NM_134013.3	Psme4	1.12	0.434	0.87	0.367
NM_011664.3	Ubb	1.14	0.133	0.96	0.737
XM_284425.1	Uba52	1.00	0.986	1.02	0.955
NM_011965.2	Psma1	1.02	0.911	1.00	0.989
NM_001033865.1	Rps27a	1.02	0.924	1.09	0.805
Glycolysis					
NM_145614.3	DLAT	0.99	0.966	1.04	0.914
NM_010699.1	Ldha	1.03	0.789	1.04	0.783
NM_011079.2	Phkg1	0.93	0.561	0.94	0.881
NM_009415.1	Tpi1	0.93	0.613	1.09	0.724
NM_023418.2	Pgam1	0.98	0.877	1.02	0.959
NM_018870.2	Pgam2	0.87	0.297	1.03	0.942
ATP synthesis					
NM_007505.2	Atp5a1	1.02	0.953	1.00	1.000
NM_198415.2	Ckmt2	1.10	0.404	0.95	0.805
NM_028388.1	Ndufv2	0.99	0.960	0.98	0.967
NM_026255.4	Slc25a6/Slc25a26	1.00	1.000	0.95	0.800
NM_145518.1	Ndufs1	0.99	0.955	1.04	0.817
NM_008618.2	Mdh1	1.01	0.970	0.94	0.825
Other					
NM_011830	IMPDH2	0.99	0.983	1.03	0.968
NM_024188.5	Oxct1	1.17	0.163	0.81	0.043*
Transcription					
NM_009372.2	Tgif1	1.22	0.285	0.69	0.151
NM_019739	Foxo1	1.73	0.021*	0.76	0.567
XM_001478948.1	Ezh1	1.06	0.585	0.96	0.884
NM_009716.2	Atf4	1.00	0.995	0.95	0.931
NM_008416.1	Junb	0.98	0.979	1.08	0.941
Translation					
XR_033381.1	Sat	1.11	0.518	0.95	0.903
NM_007918.3	Eif4ebp1	1.14	0.601	1.11	0.844
NM_013506	Eif4a2	0.98	0.908	1.01	0.984
AK019693	Eif4g3	1.00	0.987	0.96	0.850
NM_027204.2	Mrpl12	0.87	0.171	1.16	0.125
Extracellular matrix					
NM_008495.1	Lgals1	0.95	0.888	1.06	0.924
NM_015784.2	OSF-2/Postn	1.01	0.953	0.97	0.868
NM_007742.2	Col1a1	0.85	0.401	0.76	0.792
NM_007737.2	Col5a2	0.98	0.795	1.03	0.884
NM_007993	Fbn1	0.79	0.491	1.05	0.964
NM_010233.1	Fn1	0.96	0.872	0.82	0.326
Miscellaneous					
NM_013602.2	Mt1	1.11	0.774	1.15	0.696
NM_019930.1	RANBP9	1.19	0.213	0.95	0.857
NM_009974.2	Csnk2a2	0.91	0.461	1.03	0.940
NM_016792	TXNL	1.01	0.969	1.00	0.993
NM_013494.2	CPE	1.02	0.937	0.90	0.449
NM_013645.3	Pvalb	0.87	0.208	1.02	0.948
NM_008409.2	Itm2a	0.92	0.736	1.26	0.192
NM_053078.3	Nrep/P311	0.88	0.672	0.73	0.199

No common changes in muscle were observed due to doxorubicin or sACVR2B-Fc on atrogenes involved in protein degradation, energy production, growth transcription/translation, genes coding extracellular matrix proteins or other genes. FC = fold change and *P* adj. = adjusted p-value using the Benjamini and Hochberg (false discovery rate, FDR) method. *P < 0.05.
