# Supplementary material for: Systemic blockade of ACVR2B ligands prevents chemotherapy-induced muscle wasting by restoring muscle protein synthesis without affecting oxidative capacity or atrogenes
Source: Sci Rep. 2016 Sep 26;6:32695. doi: 10.1038/srep32695 (PMC5036092; doi:10.1038/srep32695)
Supplement: Supplementary Information [file srep32695-s1.pdf]

Systemic blockade of ACVR2B ligands prevents chemotherapy-induced muscle wasting by restoring muscle protein synthesis without affecting oxidative capacity or atrogenes

Nissinen TA[1], Degerman J[2], Räsänen M[2], Poikonen AR[1], Koskinen S[3], Mervaala E[4], Pasternack A[5], Ritvos O[5,6], Kivelä R[2], Hulmi JJ[1,6*].

[1]Department of Biology of Physical Activity, Neuromuscular Research Center, University of Jyväskylä, Jyväskylä, Finland

[2]Wihuri Research Institute and Translational Cancer Biology Program, University of Helsinki, Helsinki, Finland

[3]LIKES Research Center for Sport and Health Sciences, Jyväskylä, Finland

[4]Department of Pharmacology, Faculty of Medicine, University of Helsinki, Helsinki, Finland

[5]Department of Bacteriology and Immunology, Haartman Institute, University of Helsinki, Helsinki, Finland

[6]Department of Physiology, Faculty of Medicine, University of Helsinki, Helsinki, Finland

*Correspondence to juha.hulmi@jyu.fi

Supplementary information

Supplementary methods

qPCR. Three housekeeping genes (Gapdh, 36b4 and Rn18s) were analysed of which the most stable in both PCR and microarray analysis (36b4) was used for normalization. The used primers are listed below (Supplementary Table S1). MuRF1 primers were kindly provided by Dr Maarit Lehti.

Supplementary Table S1. The sequences of primer pairs used in real-time qPCR.

Transcript	Strand	Sequence, 5'-3'
36B4	Forward	GGCCCTGCACTCTCGCTTTC
36B4	Reverse	TGCCAGGACGCGCTTGT
MuRF1	Forward	GCTCAGAGAGCAGGGACTAG
MuRF1	Reverse	AAAGCACCAAATTGGCATAC
REDD1 (DDIT4)	Forward	GCCTCTGGGATCGTTTCTCG
REDD1 (DDIT4)	Reverse	GGTCAAGGCCCTCTTCTCTG
GAPDH	Forward	AACTTTGGCATTGTGGAAGG
GAPDH	Reverse	GGATGCAGGGATGATGTTCT
PGC1α_ex1a	Forward	CATGTGCAGCCAAGACTCTG
PGC1α_ex1a	Reverse	ACACCACTTCAATCCACCCA
PGC1α_ex1b	Forward	CCATGGATTCAATTTTGAAATGTG
PGC1α_ex1b	Reverse	GTTCGCAGGCTCATTGTTGT
PGC1α_ex1c	Forward	AAGTGAGTAACCGGAGGCATTC
PGC1α_ex1c	Reverse	TTCAGGAAGATCTGGGCAAAGA
PGC-1β	Forward	CAGGGTGGGGACTCTGGA
PGC-1β	Reverse	GAAGAGCTCGGAGTCATCGG
Rn18S	Forward	GCAATTATTCCCCATGAACG
Rn18S	Reverse	GGCCTCACTAAACCATCCAA
trunc-PGC-1α	Forward	TCACACCAAACCCACAGAAA
trunc-PGC-1α	Reverse	CTGGAAGATATGGCACAT

Microarray. The data analysis was performed as previously described[1]. The differentially expressed genes in the microarray data between were detected using Chipster software and the raw p-values were adjusted using the Benjamini and Hochberg (false discovery rate, FDR) method. The genes with an adjusted $P < 0.05$ and with absolute fold change ≥ 1.5 were considered to be differentially expressed. Enrichment of functionally related genes was first performed using a non-biased method by Gene Set Enrichment Analysis software (GSEA; Version 2.0)[2] as earlier[1]. We generated an atrogene gene set of "common atrogenes" in many murine atrophied muscles[3]. The collection used was the Canonical Pathways, Biocarta, KEGG and Reactome (http://www.broadinstitute.org/gsea/msigdb/collections.jsp). The number of permutations by gene set was set to 1000 and gene sets with at least 10 and no more than 500 genes were taken into account in each analysis. Each analysis was carried out five times and all the results were averaged into a single value.

Antibodies used in western blotting. To study phosphorylation of proteins of interest, antibodies recognizing phosphorylated Akt at Ser473, rpS6 at Ser240/244, p70S6K1 at Thr389, ERK1/2 MAPK at Thr202/Tyr204, AMPKα at Thr172, eIF2α at Ser51, 4E-BP1 at Thr37/46, FoxO1 at Ser256 (Cell Signaling Technology) were used. In addition, total proteins of Akt, rpS6, p70S6K1, ERK1/2, AMPK, eIF2α, 4E-BP1 and FoxO1 were analysed using specific antibodies (Cell Signaling Technology). This was conducted by reprobing the membrane after careful stripping of the membrane with Restore Western Blot Stripping Buffer (Pierce/ThermoFisher Scientific). The same reprobing protocol was used to detect GAPDH (Abcam) that was used as a loading control along with Ponceau S staining. Moreover, specific antibodies against LC3 (Sigma Aldrich), calpain1 (Cell Signaling Technology), PGC-1α (C-terminal, Calbiochem), cytochrome *c* (Santa Cruz Biotechnology), porin/VDAC1 (Calbiochem) were used to study these proteins. Additionally, relative levels of the mitochondrial OXPHOS complexes were analysed using an antibody cocktail against subunits of the five OXPHOS complexes (Abcam). Ubiquitinated proteins were detected with a horseradish peroxidase-conjugated anti-ubiquitin antibody (Santa Cruz Biotechnology). Finally, to analyse puromycin incorporation, the mouse monoclonal anti-puromycin antibody (clone 12D10, a generous gift from Dr Philippe Pierre) was used.

Associations between the measured variables. A computationally determined network was created between different variables. For this, a Katiska/Himmeli software using in GNU Octave program environment (http://www.finndiane.fi/software/katiska/) was used[4].

References

1. Kainulainen, H. *et al*. Myostatin/activin blocking combined with exercise reconditions skeletal muscle expression profile of mdx mice. *Mol. Cell. Endocrinol.* **399**, 131-142 (2015).

2. Subramanian, A. *et al*. Gene set enrichment analysis: a knowledge-based approach for interpreting genome-wide expression profiles. *Proc. Natl. Acad. Sci. U. S. A.* **102**, 15545-15550 (2005).

3. Sacheck, J. M. *et al*. Rapid disuse and denervation atrophy involve transcriptional changes similar to those of muscle wasting during systemic diseases. *FASEB J.* **21**, 140-155 (2007).

4. Makinen, V. P. *et al*. Network of vascular diseases, death and biochemical characteristics in a set of 4,197 patients with type 1 diabetes (the FinnDiane Study). *Cardiovasc. Diabetol.* **8**, 54-2840-8-54 (2009).

Supplementary Figures

Figure legends

Figure S1. Doxorubicin administration resulted in decreased muscle weights that were restored by sACVR2B-Fc treatment. Absolute tissue weights of TA (a), gastrocnemius (GA) (b) and soleus (c) muscles and epididymal fat pads (d). The figure 1 expressed these relative to tibial length, which did not differ between the groups (e). N sizes are depicted in the bar graphs. Data are presented as mean ± SEM. **P* < 0.05; ***P* < 0.01; ****P* < 0.001 (Bonferroni).

Figure S2. Gene set enrichment analysis from micro array data of gene sets of Common atrogenes (a-b) and proteasome (c-d) after single acute doxorubicin administration.

Figure S3. Changes in phosphorylated (Ser26) and total FoxO1 protein relative to Dox (a), Murf1 mRNA by qPCR relative to Ctrl (b), p-Akt(Ser473)/total Akt (c) and p-4EBP1(Thr37/46)/4EBP1 relative to Dox (d) after a single acute doxorubicin administration. N sizes are depicted in the bar graphs. Data are presented as mean ± SEM. **P* < 0.05, ***P* < 0.01 (Mann-Whitney U (a, c, d); Bonferroni (b)).

Figure S4. Gene set enrichment analysis from micro array data of gene sets of autophagy (a-b) and apoptosis (c-d) after single acute doxorubicin administration.

Figure S5. Doxorubicin did not affect electron transport chain proteins or PGC-1α protein. Contents of porin/VDAC1 protein (a), electron transport chain subunits (b–f) and PGC-1α protein (g) relative to Dox in TA muscle analysed with Western blotting. Representative blots for porin/VDAC1 and PGC-1α (h). N sizes are depicted in the bar graphs. Data are presented as mean ± SEM. **P* < 0.05, ***P* < 0.01, ****P* < 0.001 (Mann-Whitney U).

Figure S6. No changes in PGC-1α mRNA after 4 weeks experiment. mRNA expression of PGC1-1α splice variants derived from exon 1a (a), exon 1b (b) and exon 1c (c), and total N-truncated PGC-1α (d). N sizes are depicted in the bar graphs. Data are presented as mean ± SEM. No differences between the groups (Bonferroni).

Figure S7. Changes in blood haemoglobin and haematocrit at 2 weeks (a, b) and 4 weeks (c, d) of doxorubicin administration. N sizes are depicted in the bar graphs. Data are presented as mean ± SEM. $^{**}P < 0.01$ (Bonferroni).

Figure S8. Changes in body and tissue masses in response to tumour (LLC), doxorubicin or sACVR2B-Fc. Body weights (a, b) and masses of TA (c), GA (d), epididymal fat (e) and tumour (f) normalized to tibial length (TL) as well as changes in lean (g) and fat (h) mass. The cumulative dose of doxorubicin was only 12 mg/kg compared with 15–24 mg/kg in the experiments in non-tumour-bearing mice. N sizes are depicted in the bar graphs. Data are presented as mean ± SEM. $^{*}P < 0.05$, $^{**}P < 0.01$, $^{***}P < 0.001$ (Bonferroni), except for Fig c, in which different letter (a, b) denotes a significant difference ($P < 0.05$, Bonferroni).

Figure S9. Correlations. mTORC1 and protein synthesis were increased in response to sACVR2B-Fc while REDD1 was acutely increased in response to a single injection of doxorubicin. The first figure thus includes Dox and Dox + sACVR2Bgroups while the second one control mice and doxorubicin-injected mice. These correlations were statistically significant ($P = 0.013$ and $P = 0.034$, respectively).

Fig. S1

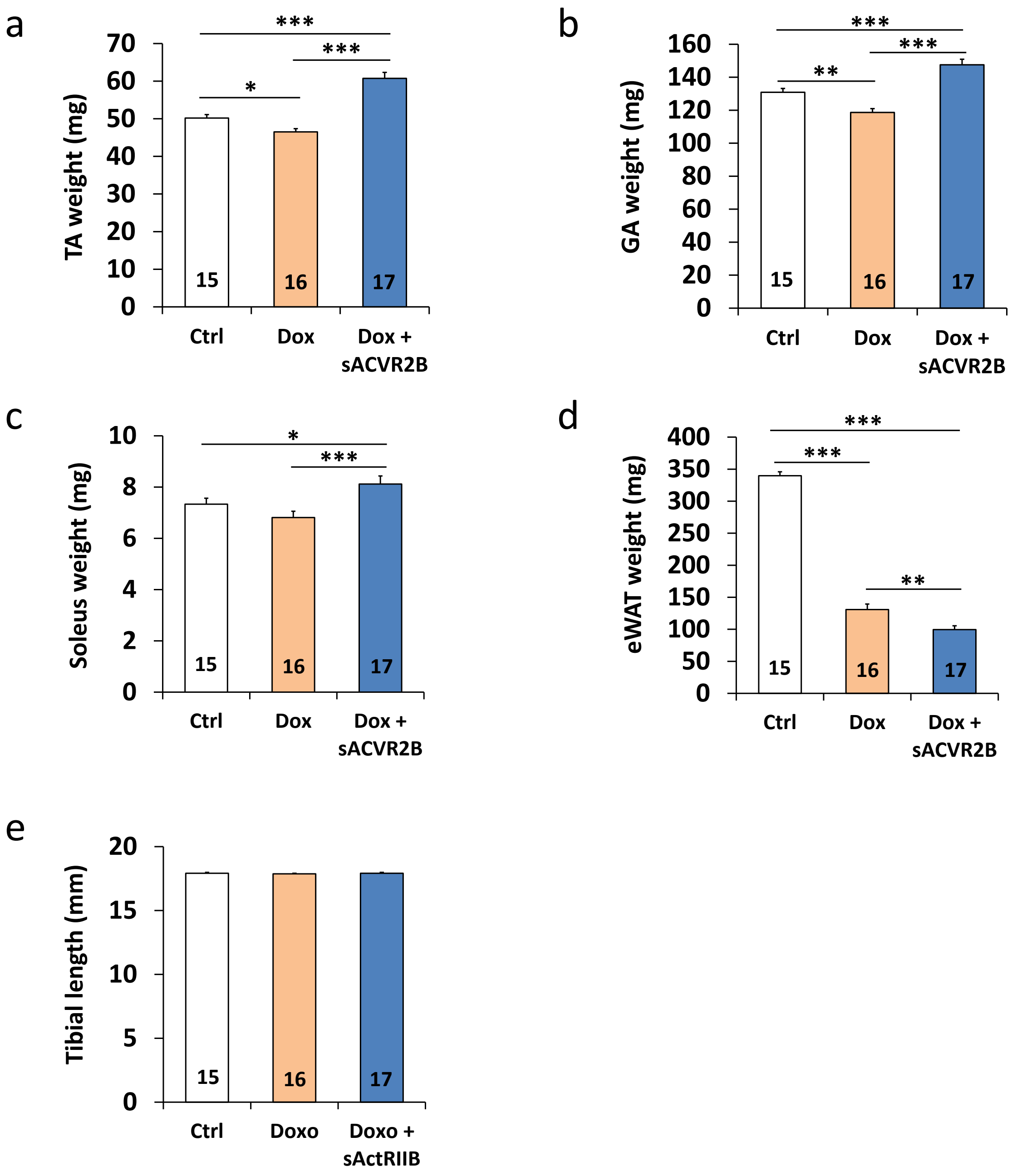

a
TA weight (mg)
70
60
50
40
30
20
10
0
***
***
*
15
16
17
Ctrl
Dox
Dox + sACVR2B
b
GA weight (mg)
160
140
120
100
80
60
40
20
0
***
***
**
15
16
17
Ctrl
Dox
Dox + sACVR2B
c
Soleus weight (mg)
10
8
6
4
2
0
*
***
15
16
17
Ctrl
Dox
Dox + sACVR2B
d
eWAT weight (mg)
400
350
300
250
200
150
100
50
0
***
***
**
15
16
17
Ctrl
Dox
Dox + sACVR2B
e
Tibial length (mm)
20
15
10
5
0
15
16
17
Ctrl
Doxo
Doxo + sActRIIB

Fig. S2

Common Atrogenes

a Genes regulated (Dox vs. control)

b Genes regulated (Dox + sACVR2B vs. Dox)

Upregulated

Downregulated

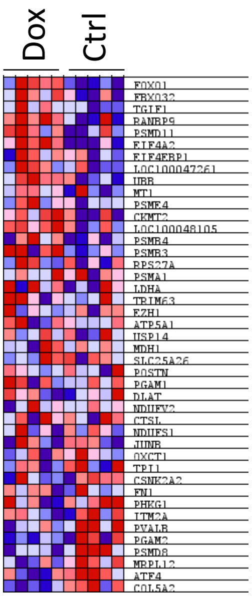

FDR = 0.050, NES 1.44

(Single gene set: nominal FDR)

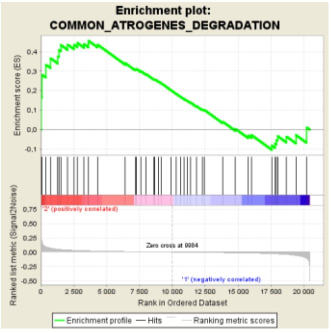

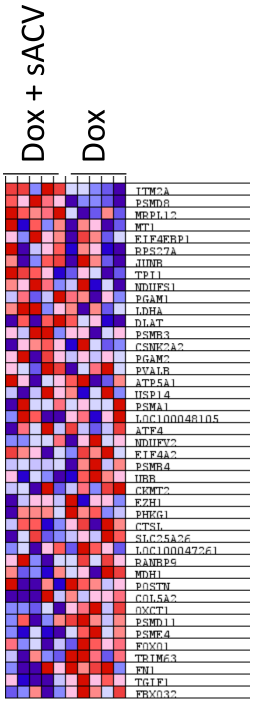

FDR = 0.020, NES -1.61

(Single gene set: nominal FDR)

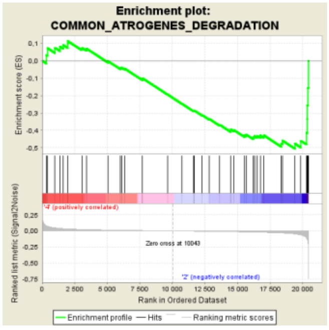

KEGG PROTEASOME

c Genes regulated (Dox vs. Ctrl)

d Genes regulated (Dox + sACVR2B vs. Dox)

Upregulated

Downregulated

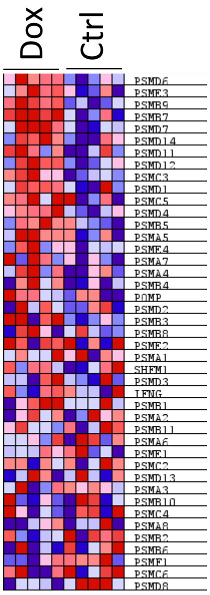

FDR = 0.041, NES 1.79

Ns. (FDR = 0.601, NES -1.16)

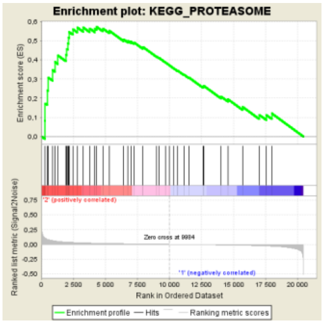

Fig. S3

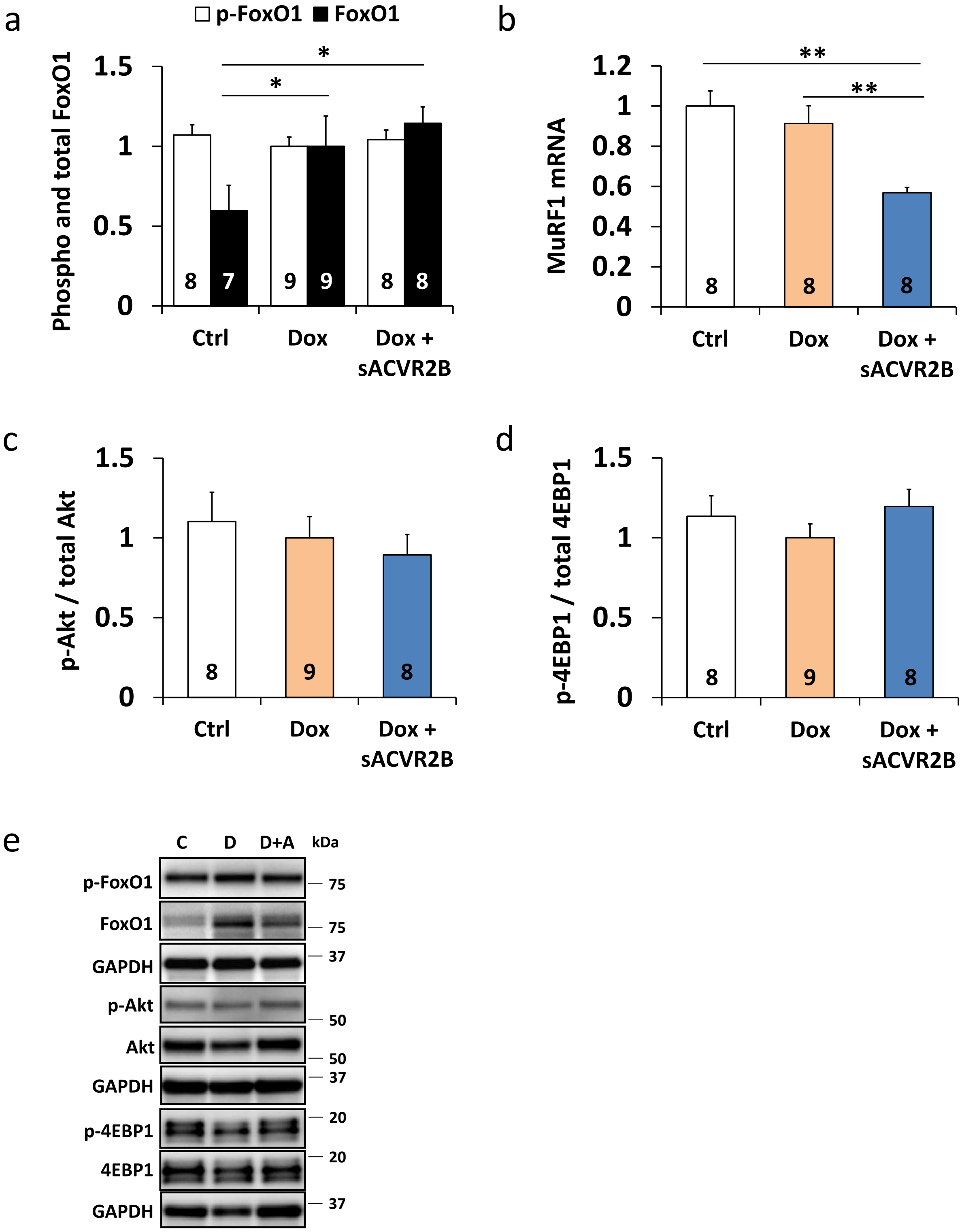

a
p-FoxO1
FoxO1
Phospho and total FoxO1
1.5
1
0.5
0
*
*
8
7
9
9
8
8
Ctrl
Dox
Dox + sACVR2B
b
MuRF1 mRNA
1.2
1
0.8
0.6
0.4
0.2
0
**
**
8
8
8
Ctrl
Dox
Dox + sACVR2B
c
p-Akt / total Akt
1.5
1
0.5
0
8
9
8
Ctrl
Dox
Dox + sACVR2B
d
p-4EBP1 / total 4EBP1
1.5
1
0.5
0
8
9
8
Ctrl
Dox
Dox + sACVR2B
e
C
D
D+A
kDa
p-FoxO1
75
FoxO1
75
GAPDH
37
p-Akt
50
Akt
50
GAPDH
37
p-4EBP1
20
4EBP1
20
GAPDH
37

Fig. S4

KEGG REGULATION OF AUTOPHAGY

a Genes regulated (Dox vs. Ctrl)

b Genes regulated (Dox + sACVR2B vs. Dox)

Upregulated

Downregulated

Ns. (FDR = 0.477, NES 1.15)

Ns. (FDR = 0.477, NES -1.39)

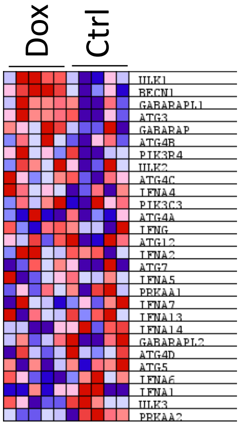

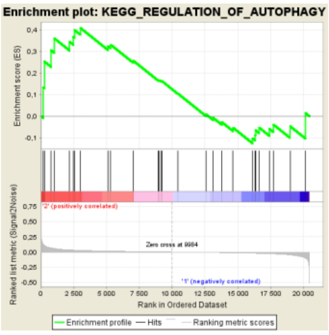

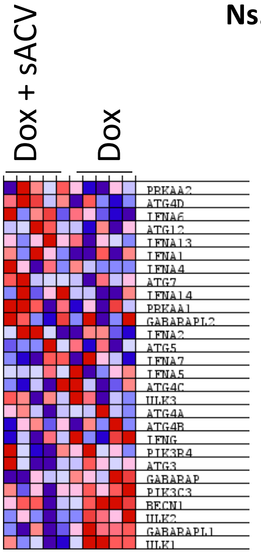

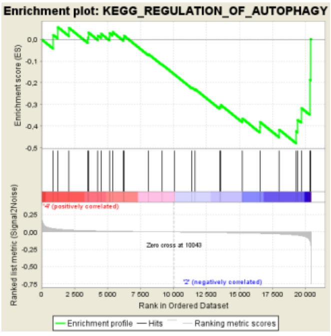

REACTOME INTRINSIC PATHWAY FOR APOPTOSIS

c Genes regulated (Dox vs. Ctrl)

d Genes regulated (Dox + sACVR2B vs. Dox)

Upregulated

Downregulated

FDR = 0.011, NES 1.93

Ns. (FDR = 0.601, NES -1.16)

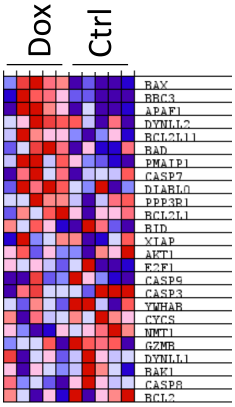

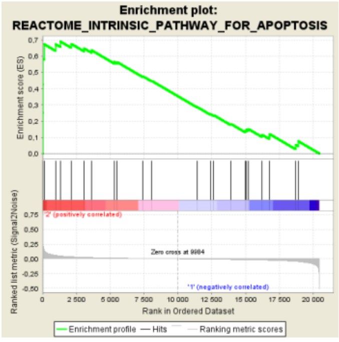

Fig. S5

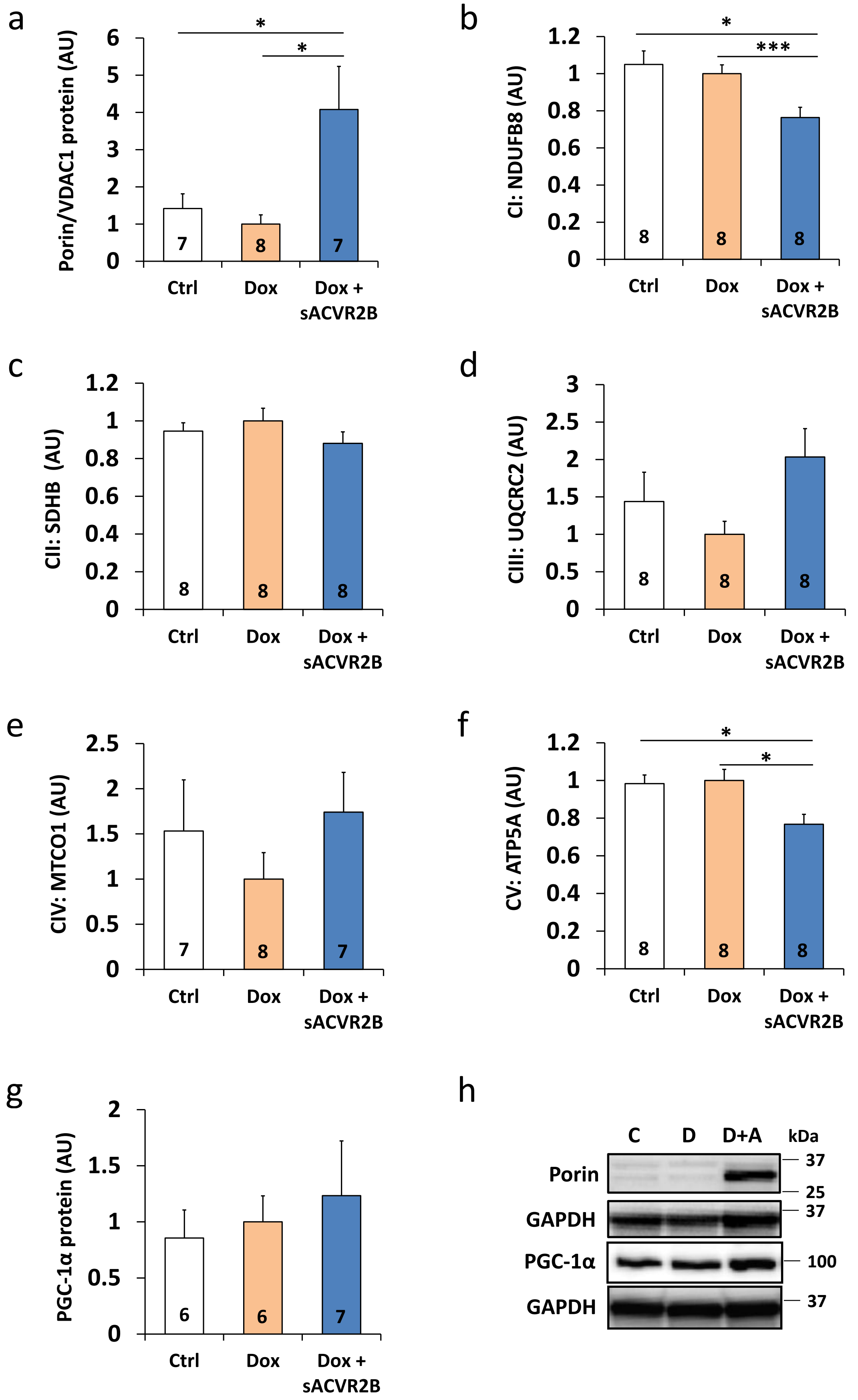

a
Porin/VDAC1 protein (AU)
Ctrl
Dox
Dox + sACVR2B
b
CI: NDUFB8 (AU)
c
CII: SDHB (AU)
d
CIII: UQCRC2 (AU)
e
CIV: MTCO1 (AU)
f
CV: ATP5A (AU)
g
PGC-1α protein (AU)
h
C
D
D+A
kDa
Porin
GAPDH
PGC-1α
GAPDH
37
25
37
100
37

Fig. S6

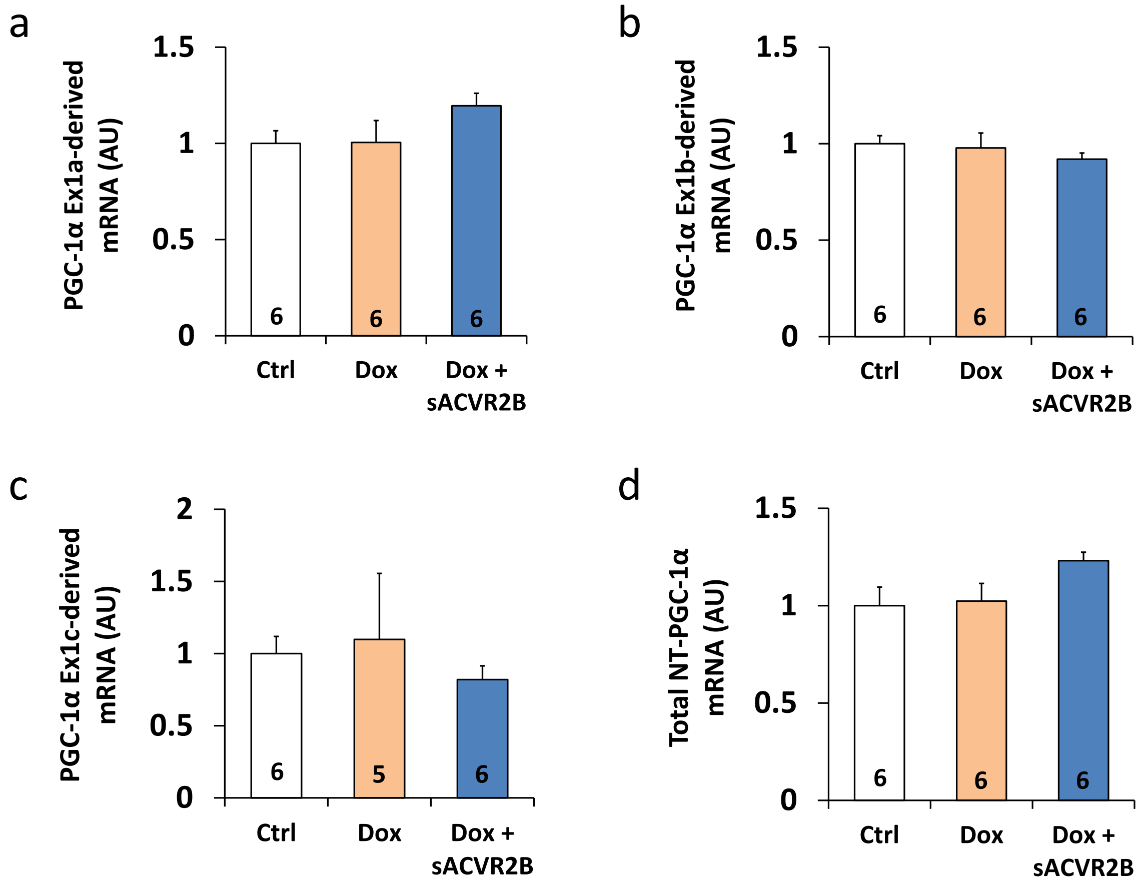
a
PGC-1α Ex1a-derived mRNA (AU)
1.5
1
0.5
0
6
6
6
Ctrl
Dox
Dox + sACVR2B
b
PGC-1α Ex1b-derived mRNA (AU)
1.5
1
0.5
0
6
6
6
Ctrl
Dox
Dox + sACVR2B
c
PGC-1α Ex1c-derived mRNA (AU)
2
1.5
1
0.5
0
6
5
6
Ctrl
Dox
Dox + sACVR2B
d
Total NT-PGC-1α mRNA (AU)
1.5
1
0.5
0
6
6
6
Ctrl
Dox
Dox + sACVR2B

Fig. S7

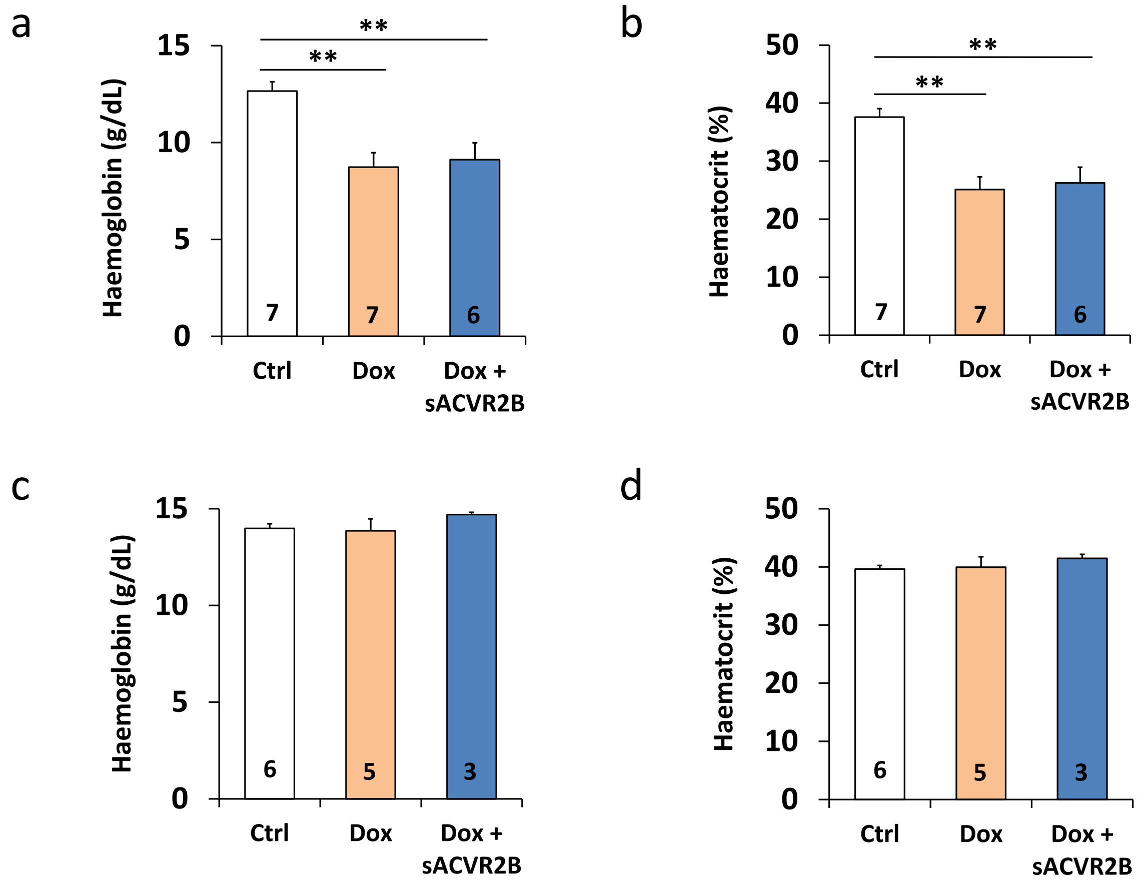
a
b
c
d
Haemoglobin (g/dL)
Haematocrit (%)
15
10
5
0
50
40
30
20
10
**
7
6
5
3
Ctrl
Dox
Dox +
sACVR2B

Fig. S8

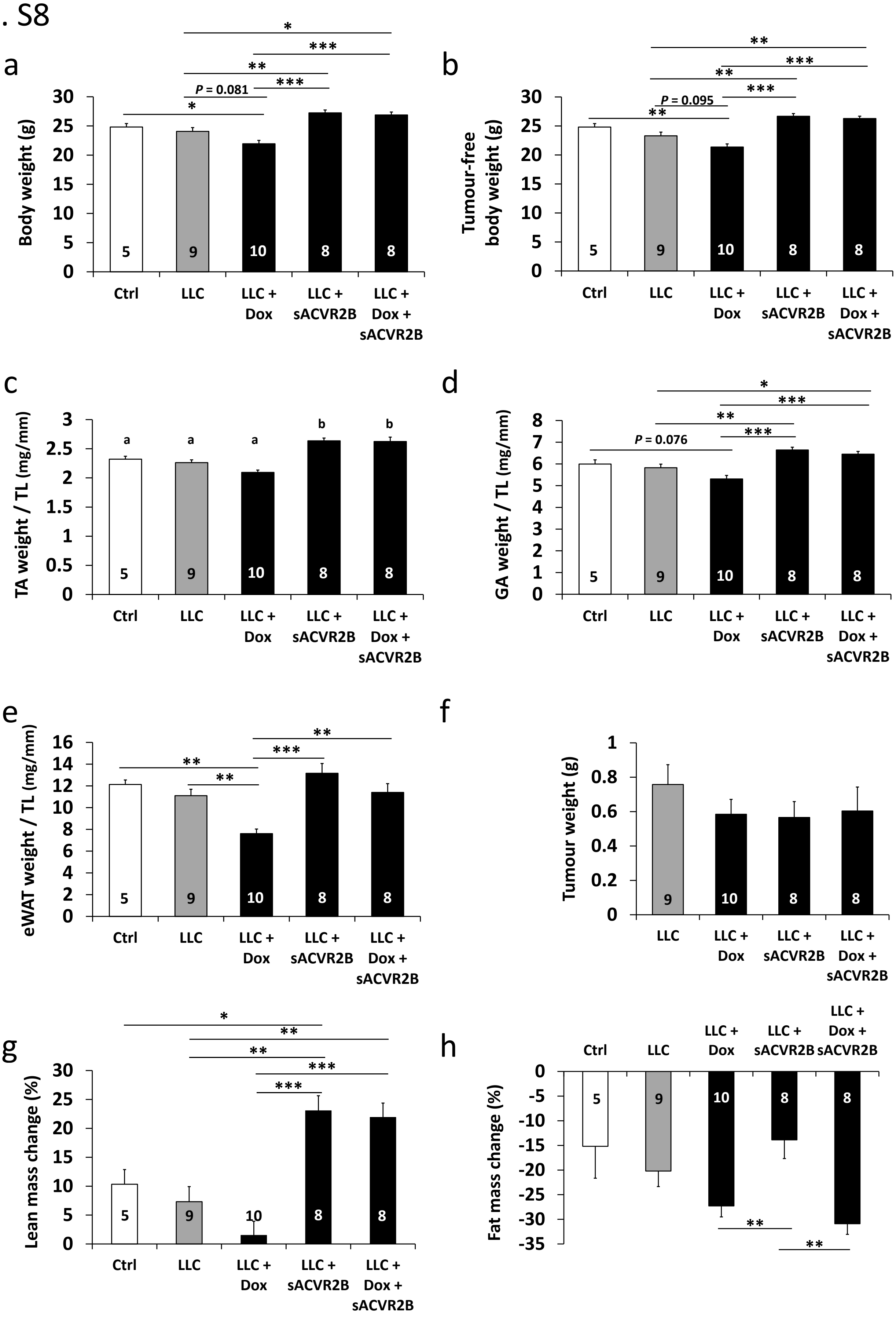

a
Body weight (g)
P = 0.081
b
Tumour-free body weight (g)
P = 0.095
c
TA weight / TL (mg/mm)
d
GA weight / TL (mg/mm)
P = 0.076
e
eWAT weight / TL (mg/mm)
f
Tumour weight (g)
g
Lean mass change (%)
h
Fat mass change (%)
Ctrl
LLC
LLC + Dox
LLC + sACVR2B
LLC + Dox + sACVR2B

Fig. S9

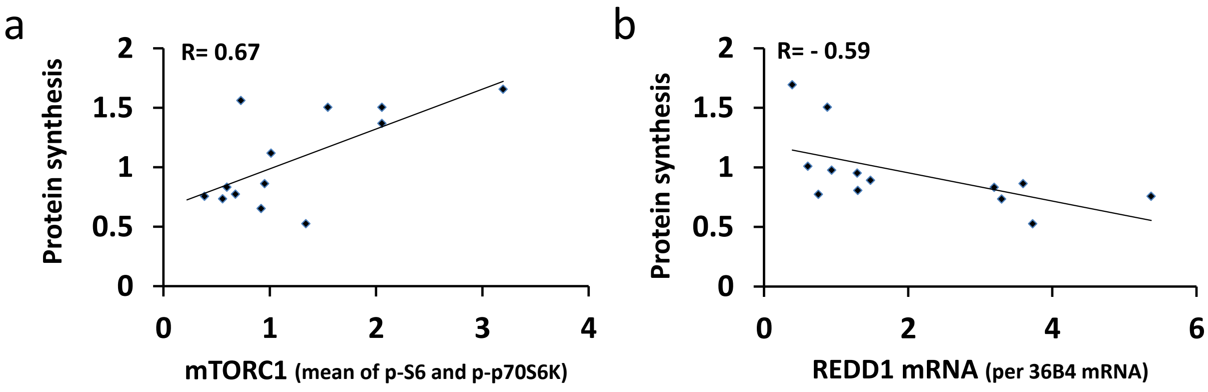
a
R= 0.67
Protein synthesis
mTORC1 (mean of p-S6 and p-p70S6K)
0
0.5
1
1.5
2
0
1
2
3
4
b
R= - 0.59
Protein synthesis
REDD1 mRNA (per 36B4 mRNA)
0
0.5
1
1.5
2
0
2
4
6
